# *Annona muricata* L. peel extract inhibits carbohydrate metabolizing enzymes and reduces pancreatic β-cells, inflammation, and apoptosis via upregulation of PI3K/AKT genes

**DOI:** 10.1371/journal.pone.0276984

**Published:** 2022-10-27

**Authors:** Oluwafemi Adeleke Ojo, Susan Grant, Jennifer Chidubem Amanze, Abosede Itunuoluwa Oni, Adebola Busola Ojo, Tobiloba Christiana Elebiyo, Tajudeen Olabisi Obafemi, Damilare Iyinkristi Ayokunle, Akingbolabo Daniel Ogunlakin

**Affiliations:** 1 Department of Biochemistry, Landmark University, Omu-Aran, Nigeria; 2 Department of Biochemistry, Ekiti State University, Ado-Ekiti, Nigeria; 3 Department of Biochemistry, Afe Babalola University, Ado-Ekiti, Nigeria; 4 Department of Pure and Applied Biology, Bowen University, Iwo, Nigeria; 5 Department of Biochemistry, Bowen University, Iwo, Nigeria; Amity University, INDIA

## Abstract

**Background and objective:**

*Annona muricata* L. peel has been recognized for many ethnobotanical uses, including diabetes management. However, limited detailed scientific information about its mechanism of antidiabetic activity exists. The objective of this study was to evaluate the anti-diabetic properties of an aqueous extract of *A*. *muricata* peel (AEAMP) and its mechanism of action on alloxan-induced diabetic rats.

**Methods:**

*In vitro* antidiabetic assays, such as α-amylase and α-glucosidase were analyzed on AEAMP. Alloxan monohydrate (150 mg/kg b.w) was used to induce diabetes in the rats. 150 mg/kg b.w positive control group doses of 6.67, 13.53, and 27.06 mg/kg were administered to 3 groups for twenty-one days. The positive control group was administered 30 mg/kg of metformin. The negative and normal control groups were administered distilled water. The fasting blood glucose, serum insulin, lipid profile, inflammatory cytokines, antioxidant markers, carbohydrate metabolizing enzymes, and liver glycogen were analyzed as well as PI3K/AKT and apoptotic markers PCNA and Bcl2 by RT-PCR.

**Results:**

AEAMP inhibited α-amylase and α-glucosidase enzymes more effectively than acarbose. AEAMP reduced FBG levels, HOMA-IR, G6P, F-1,6-BP, MDA, TG, TC, AI, CRI, IL-6, TNF-α, and NF-κB in diabetic rats. Furthermore, in diabetic rats, AEAMP improved serum insulin levels, HOMA-β, hexokinase, CAT, GST, and HDL-c. Liver PI3K, liver PCNA and pancreas PCNA were not significantly different in untreated diabetic rats when compared to normal rats suggesting alloxan induction of diabetes did not downregulate the mRNA expression of these genes. AEAMP significantly up-regulated expression of AKT and Bcl2 in the liver and pancreatic tissue. It is interesting that luteolin and resorcinol were among the constituents of AEAMP.

**Conclusions:**

AEAMP can improve β-cell dysfunction by upregulating liver AKT and pancreatic PI3K and AKT genes, inhibiting carbohydrate metabolizing enzymes and preventing apoptosis by upregulating liver and pancreatic Bcl2. However, the potential limitation of this study is the unavailability of equipment and techniques for collecting more data for the study.

## Introduction

Diabetes is a long-term health condition and has been ranked as the 9^th^ leading cause of adult mortality globally [1]. In 2014, it was estimated that five to ten percent of the world’s 386 million diabetics had type 1 diabetes (T1DM) [2–4]. T1DM is characterized by insulin deficiency due to pancreatic β-cell loss, leading to hyperglycaemia. The hallmark of T1DM is the destruction of the pancreas, which leads to the non-secretion of insulin required to maintain normal blood glucose levels [5].

One of the recognized pathologies of T1DM is an autoimmune disease that leads to the destruction of insulin-producing pancreatic beta cells over months or years [6]. Damage to beta-cells can result in high blood glucose, a prevalent metabolic condition involving carbohydrates, lipids, and proteins that is rapidly becoming an epidemic [7]. Insulin deficiency leads to hyperglycemia and ketosis. Chronic hyperglycemia is associated with long-term damage, leading to dysfunction of the kidney, eyes, nerves, heart, and blood vessels [8]. Based on statistics, 2.8% of the world’s population currently has diabetes, with that proportion anticipated to rise to around 5.4% by 2045 [7]. The high prevalence, varied pathogenesis, and complications of the disease are pointers to the urgent need for effective treatment options [9]. In recent times, different treatments, like insulin, drug administration, and food therapy, have recently been effective in T1DM management [10].

Many oral medications exert anti-diabetic effects via various mechanisms [11, 12]. Metformin, imatinib, gliptines, α-glucosidase inhibitors, and gliflozines are some of the drugs used to treat T1DM. However, further studies are necessary for conclusive validation of their efficacy and safety. Recent evidence-based investigations did not necessarily confirm the efficacy and safety of these oral medications as adjuvants for T1DM patients. Thus, they still signify off-label medication for its management [11, 12]. Many treatment regimens with the exploitation of medicinal plants are recommended for diabetes. *Allium sativum*, *Azadirachta indica*, *Blighia sapida*, and *Caesalpinia bonducella* are a few of the hundreds of plants documented to have produced positive anti-diabetic effects [13–18].

*Annona muricata* L. (*A*. *muricata*) is a medicinal plant in the *Annonaceae* family and is recognized for its numerous traditional benefits [19, 20]. *Annona* is utilized in traditional medicine in its entirety. It is believed to be a rich source of antioxidants for several ailments [19–21]. The peels are traditionally used among Abia villagers in Nigeria to treat diabetes, headaches, and liver complications. The efficacy of most medicinal plants has been linked to their constituents [22, 23]. There is, though, limited detailed information linking the antidiabetic activity of the *A*. *muricata* peel to a mechanism of action. Thus, the study examined the antidiabetic activity of an aqueous extract of *A*. *muricata* peel (AEAMP) in an alloxan-induced diabetic rat model and also determined the mechanisms of action of this extract. The objective of this study is to examine the role of *A*. *muricata* peel (AEAMP) in abrogating alloxan-induced diabetes via suppressing oxidative stress, inflammation, and β-cell apoptotic death, and by upregulating glucose uptake by stimulating the PI3K/AKT genes. Furthermore, to identify potential bioactive compounds present in the plant that might be responsible for the antidiabetic activity observed and to understand the mechanisms by which potential mRNA genes are upregulated or downregulated in the management of diabetes.

## Materials and methods

### Plant material

The peels of *A*. *muricata* were obtained from a local farm in Abia village, Ohafia Local Government Area, Abia State. The plant was identified at the Forestry Research Institute of Nigeria (FRIN), Nigeria, voucher number FHI 113160.

### Chemicals, apparatus, and general procedures

All of the chemicals were of analytical grade. Methanol, phosphoric acid, vanillin, cyanidin, and benzoic acid were purchased from Merck (Darmstadt, Germany). Quercetin, rescorcinol, and luteolin were acquired from Sigma Chemical Co. (St. Louis, MO, USA). High performance liquid chromatography (HPLC-DAD) was performed with a Shimadzu Prominence Auto Sampler (SIL-20A) HPLC system (Shimadzu, Kyoto, Japan), equipped with Shimadzu LC-20AT reciprocating pumps connected to a DGU 20A5 degasser with a CBM 20A integrator, SPD-M20A diode array detector, and LC solution 1.22 SP1 software.

### Preparation of aqueous extract of *A*. *muricata* peel (AEAMP)

Peels of *A*. *muricata* were sliced into little pieces and shed-dried at room temperature for 8 weeks. We obtained the aqueous extract by freeze-drying the filtrate obtained from the extraction of 50 g of the powdered material with distilled water for 48 hours.

### High-performance liquid chromatography (HPLC-DAD)

We carried out HPLC analysis of AEAMP, following the standard procedure described by Ojo *et al*. [24]. Reverse phase chromatographic analyses were carried out under gradient conditions using a C_18_ column (4.6 mm x 150 mm) packed with 5μm diameter particles; the mobile phase was water containing 1% phosphoric acid (A) and acetonitrile (B), and the composition gradient was 17% of B until 10 min and changed to obtain 20%, 30%, 50%, 60%, 70%, 20%, and 10% B at 20, 30, 40, 50, 60, 70, and 80 min, respectively. The samples and mobile phase were filtered through a 0.45 μm membrane filter (Millipore) and then degassed in an ultrasonic bath prior to use. The *Annona muricata* peel extract was analyzed at a concentration of 10 mg/mL. The flow rate was 0.7 mL/min, the injection volume was 50 μl and the wavelength was 327 nm for benzoic acid, cyaniding, and vanillin, and 366 nm for quercetin, resorcinol, and luteolin. Stock solutions of standards references were prepared in the HPLC mobile phase at a concentration range of 0.030–0.500 mg/mL. Chromatography peaks were confirmed by comparing their retention times with those of reference standards and by DAD spectra (200 to 600 nm). All chromatographic operations were carried out at ambient temperature and in triplicate.

### Evaluation of α-amylase activity

We used the method described by Shai et al. [25] to assess AEAMP’s inhibitory activity against the -amylase enzyme. The samples (15–240 μg/ml) were incubated for 20 minutes at 37°C with 500 μl of porcine pancreatic amylase (2 U m/L) in 100 mmol/L of phosphate buffer (pH 6.8) in a known volume (250 μl). A known volume (250 μl) of 1% starch dissolved in 100 mmol/L phosphate buffer (pH 6.8) was added to the reaction mixture and incubated at 37°C for 1 hour. A milliliter of dinitrosalicylic acid color reagent (1%, 3,5-dinitrosalicylic acid, 0.2% phenol, 0.05% Na_2_SO_3_, and 1% sodium hydroxide) was added and heated for 10 minutes at 100°C. After cooling to 25°C in a cold water bath, the absorbance of the resulting mixture was read at 540 nm. Acarbose was used as a standard. The result was calculated and expressed as a percentage.

### Evaluation of α-glucosidase activity

We used the method described by Nguelefack et al. [26] to assess AEAMP’s inhibitory activity against the α-glucosidase enzyme. One mg of α-glucosidase (Saccharomyces cerevisiae, Sigma-Aldrich, USA) was dissolved in 100 ml of phosphate buffer (pH 6.8) containing 200 mg of bovine serum albumin. The reaction mixture, consisting of 10 μl of sample at varying concentrations (15–240 μg/ml) was premixed with 490 μl of phosphate buffer (pH 6.8) and 250 μl of 5 mM p-nitrophenyl α-D-glucopyranoside. After pre-incubating at 37°C for 15 minutes, the reaction was terminated by the addition of 2000 μl of 200 mM Na_2_CO_3_. α-Glucosidase activity was determined spectrophotometrically at 400 nm by measuring the quantity of p-nitrophenol released from p-NPG. Acarbose was used as a positive control of the α-glucosidase inhibitor.

### Dosage determination and experimental animals

Male Wistar rats (36) (240.20 ± 10.52 g) were purchased from the Department of Biochemistry, University of Ilorin. The rats were then housed in cages under standard conditions (room temperature with a 12 h light-dark cycle). The rats were acclimatized for two weeks before the experiment began. From the ethnobotanical survey conducted in an Abia village in Nigeria during the study, the mentioned human therapeutic doses of *A*. *muricata* peels are between 1 and 9 g. The dose for rats was calculated considering the human to albino rat conversion factor (conversion factor = 0.162) according to body surface area. This was done by dividing it by the adult human weight of 60 kg and multiplying it by a factor to accommodate the body surface area of the animal [27]. The calculated dose obtained falls within the range of approximately 100 mg/kg b.w rat. Therefore, dose levels of 6.76, 13.53, and 27.06 mg/kg b.w. were used in this research work.

### Induction of DM

Rats that were deprived of food pellets overnight were each treated with a single intraperitoneal injection of alloxan at a dose of 150 mg/kg [24]. Alloxan was freshly prepared by dissolving it in 0.9% normal saline. The Wistar rats were given a 150 mg/kg body weight single injection of alloxan dissolved in normal saline (NaCl) and a 5% glucose solution for 24 hours to prevent hypoglycaemic shock. The alloxan-diabetic rats’ fasting glucose levels were measured on the third day after injection, resulting in a blood glucose level of ≥ 250 mg/dL.

### Animal groups and extract treatment

Thirty-six male Wistar rats were chosen and put into a group of six containing six rats each as follows:

Group 1: Distilled water + normal rats

Group 2: Untreated diabetic rats

Group 3: Diabetic rats were given 30 mg/kg body weight of metformin.

Group 4: Diabetic rats were given 6.76 mg/kg body weight of AEAMP.

Group 5: Diabetic rats were given 13.53 mg/kg body weight of AEAMP.

Group 6: Diabetic rats were given 27.06 mg/kg body weight of AEAMP.

Following feeding, both the drug (metformin) and the extract were administered. By reconstituting the extract in water, it was supplied via oral gavage.

### Ethical approval

The Landmark University animal ethics committee accepted this work with the permission number LUAC/2021/007A.

Sample collection and analysis of organs

This study lasted three weeks, after which the rats were euthanized via halothane anesthesia; the liver and pancreas were harvested and homogenized in cold phosphate buffer (0.01M, pH 7.4, 1:5 w/v) following the procedure described by [17]. Blood was drawn from the heart and placed in a clean, dry centrifuge tube, which was then allowed to clot at 25°C before being centrifuged at 5,000 rpm for 20 minutes to preserve the sera for further analysis. The tissues harvested were centrifuged for 15 min at 10,000 rpm, and the supernatant was separated with Pasteur pipettes, transferred into specimen bottles, and frozen at -80°C for further biochemical analysis.

### Biochemistry parameters in serum and liver

We used the technique described by [28] to assess serum insulin concentration, which used an ELISA kit from England in a multiple plate ELISA reader (Winooski, Vermont, USA). We evaluated liver glycogen following the method of Murat and Serfaty [29]. Serum total cholesterol was evaluated following the procedure described by Fredrickson *et al*. [30]. We also assessed triglyceride via the procedure illustrated by Tietz [31]. The Jacob *et al*. method was employed to determine the HDL-c [32]. We estimated LDL and VLDL cholesterol by employing the technique illustrated by Friedewald *et al*. [33]. The atherogenic index (AI) was calculated by using the expression in Eq 1 [34]. We calculated the coronary artery index (CRI) by using the described expression in Eq 2 [35]. The Homeostasis model assessment of insulin resistance (HOMA-IR) and β-cell score (HOMA-β) were determined via the expression described by Wilson and Islam [36] in Eqs 3 and 4.


$$Atherogenic\;index=\frac{Total\;cholesterol-High\;density\;lipoprotein\;cholesterol}{High\;density\;lipoprotein\;cholesterol}$$ (1)

$$Coronary\;artery\;index=\frac{Low\;density\;lipoprotein\;cholesterol}{High\;density\;lipoprotein\;cholesterol}$$ (2)

$$HOMA-IR=\frac{\left[insulin\;(U/L)X\;blood\;glucose\;(mmol/L)\right]}{22.5}$$ (3)

$$HOMA-\beta =\frac{\left[20\;X\;insulin\;(U/L)\right]}{\left[blood\;glucose\;(mmol/L)-3.5\right]}$$ (4)

### Determination of antioxidant biomarkers

In this study, antioxidant biomarkers including reduced glutathione (GSH) levels, catalase, superoxide dismutase activities, and MDA levels were analyzed [37–40].

### Determination of the activities of glycolytic enzymes

We analyzed the activities of hexokinase, glucose-6-phosphatase (G6P), and fructose 1,6-bisphosphate (F-1,6-BP) on the liver supernatant [41–43].

### Evaluation of inflammatory biomarkers in rat serum

We analyzed for TNF-α, IL-6, and NF-κB, via the procedure illustrated in the ELISA kits (Biosource, USA).

### RT (reverse transcriptase) PCR (RT-PCR) analysis

#### Isolation of RNA

We isolated total RNA from the liver and pancreas following the procedure illustrated by Ojo *et al*. [24, 44]. TRIzol reagent (Zymo Research, USA, Cat: R2050-1-50, Lot: ZRC186885) was used to homogenize the liver and pancreas at 4°C. Absolute RNA was apportioned in chloroform solvent (BDH Analytical Chemicals, Poole, England, Cat: 10076-6B) and centrifuged at 15,000 rpm/15 min (Abbott Laboratories, Model: 3531, Lake Bluff, Illinois, United States). The RNA from the supernatant was precipitated using an equivalent amount of isopropanol (Burgoyne Urbidges & Co, India, Cat: 67-63-0). The RNA pellet was washed two times in 70% ethanol (70 ml of absolute ethanol (BDH Analytical Chemicals, Poole, England, Cat: 10107-7Y) in 30 ml of nuclease-free water (Inqaba Biotec, West Africa, Lot no: 0596C320, code: E476-500ML)). The pellets were air-dried for 5 min and solubilized in RNA buffer (1 mM sodium citrate, p^H^ 6.4).

#### cDNA conversion

Before cDNA transformation, absolute RNA amount (concentration (μg/ml) = 40 * A_*260*_) and quality (≥ 1.8) were evaluated using the proportion of A_260_/A_280_ (A = absorbance) read via a spectrophotometer. DNA contamination was removed from RNA following DNAse I treatment (NEB, Cat: M0303S) as itemized by the manufacturer. 2 μl solution comprising 100 ng DNA-free RNA was changed to cDNA via M-MuLV Reverse transcriptase Kit (NEB, Cat: M0253S) in 20 μl final volume (2 μl, N^9^ random primer mix; 2 μl, 10X M-MuLV buffer; 1 μl, M-MuLV RT (200 U/μl); 2 μl, 10 mM dNTP; 0.2 μl, RNase Inhibitor (40 U/μl), and 10.8 μl nuclease-free water). The reaction process continued at 25°C O/N. Inactivation of M-MuLV reverse transcriptase was achieved at 65°C/20 min.

#### PCR amplification and agarose gel electrophoresis

We used the protocol illustrated in Ojo *et al*. to perform PCR intensification for the evaluation of genes whose primers (Primer3 software) are listed below [24, 44]. In a 25 μl volume mixture of 2 μl cDNA (10 ng), 2 μl primer (100 pmol), and 2 μl water, PCR enhancement was achieved. 12.5 μl Ready Mix Taq PCR master mix (One Taq Quick-Load 2x, master mix, NEB, Cat: M0486S) and 8.5 μl nuclease-free water. An initial denaturation at 95°C for 5 minutes was followed by 20 cycles of amplification (denaturation at 95°C for 30 seconds, annealing for 30 seconds, and extension at 72°C for 60 seconds) and a final extension at 72°C for 10 minutes). In all tests, we incorporated negative controls, where the mixture has no cDNA. The amplicons were sorted out on a 1.5% agarose gel (Cleaver Scientific Limited: Lot: 14170811) in Tris (RGT reagent, China, Lot: 20170605). -Borate (JHD chemicals, China, Lot 20141117) EDTA buffer (pH 8.4). The primer information can be found in the S1 File.

#### Amplicon image processing

We caught the in-gel amplicon bands pictures on camera and treated them on the Keynote platform as revealed by Ojo *et al*. and evaluated them via image-J software [24, 44].

#### Histological studies

After paraffin embedding, the fixed pancreatic tissues were stained with hematoxylin and eosin. A Leica slide scanner was used to view the slides (SCN 4000, Leica Biosystems, Germany) [45].

### Statistical analysis

The study was conducted in triplicates for *in vitro* studies, and results are expressed as numbers, percentages, and mean ± SD. The data were analyzed using one-way analysis of variance (ANOVA) with Tukey’s post hoc test. We used the GraphPad Prism 9 edition to plot the graphs. A p value of *<* 0.05 is considered significant.

## Results

### High-performance liquid chromatography (HPLC-DAD) of AEAMP

**Fig 1:**
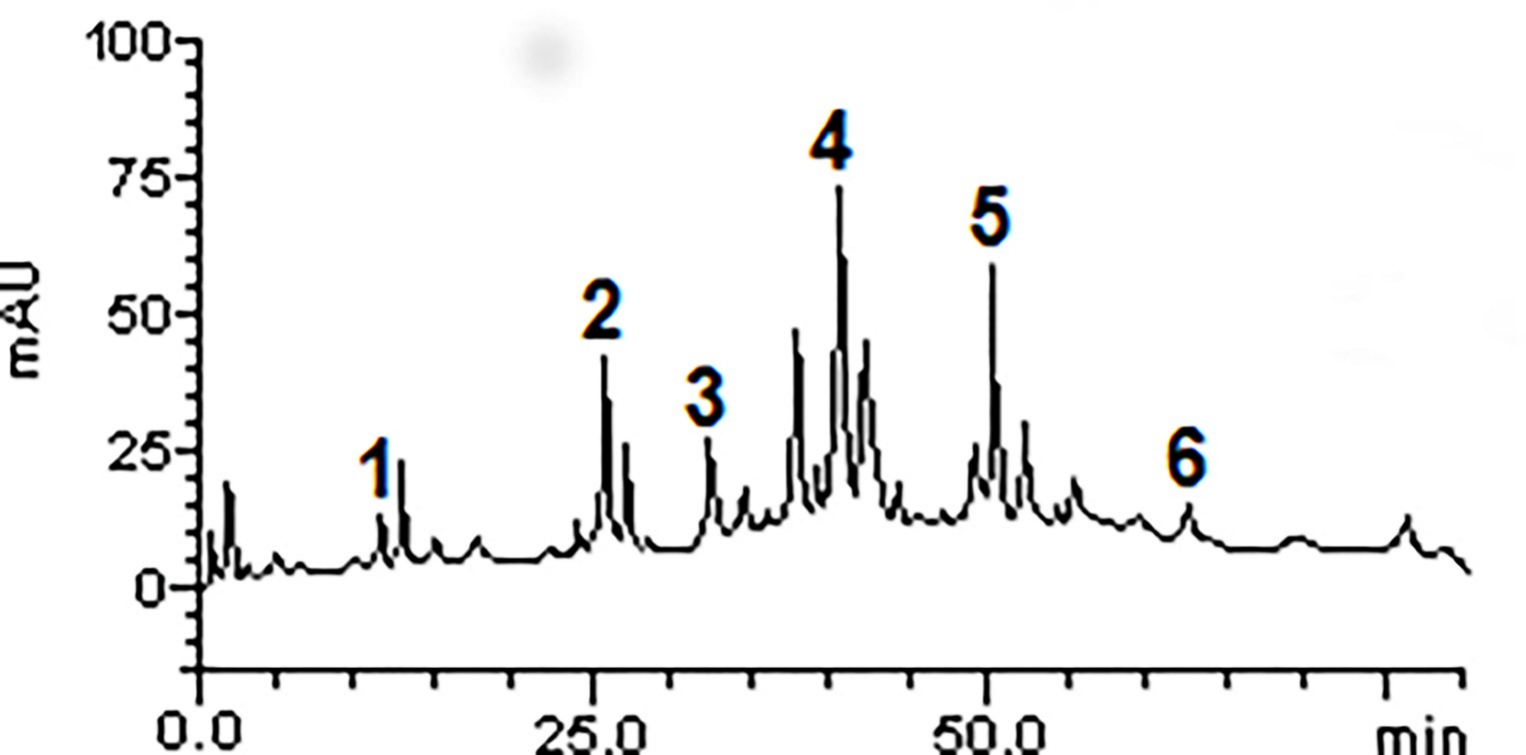
HPLC-DAD chromatogram of AEAMP. Cyanidin (peak 1), Vanillin (peak 2), Benzoic acid (peak 3), Luteolin (peak 4), Resorcinol (peak 5) and Quercetin (peak 6). AEAMP: aqueous extract of *Annona muricata* peel.

The HPLC-DAD analysis of AEAMP (Fig 1) revealed several constituents present as revealed by the chromatograms. The compounds are luteolin, resorcinol, vanillin, benzoic acid, cyanidin, and quercetin (Table 1).

**Table 1:** Bioactive compounds identified in AEAMP.

Compounds	Retention time	Concentration (μg/10g)
Cyanidin	7.198	0.4796
Vanillin	5.715	4.7474
Benzoic acid	6.315	1.4180
Luteolin	1.398	80.2484
Resorcinol	4.607	13.0368
Quercetin	7.415	0.0228

AEAMP: Aqueous extract of *Annona muricata* peels

### *In vitro* antidiabetic study (α-amylase and α-glucosidase activities)

**Fig 2:**
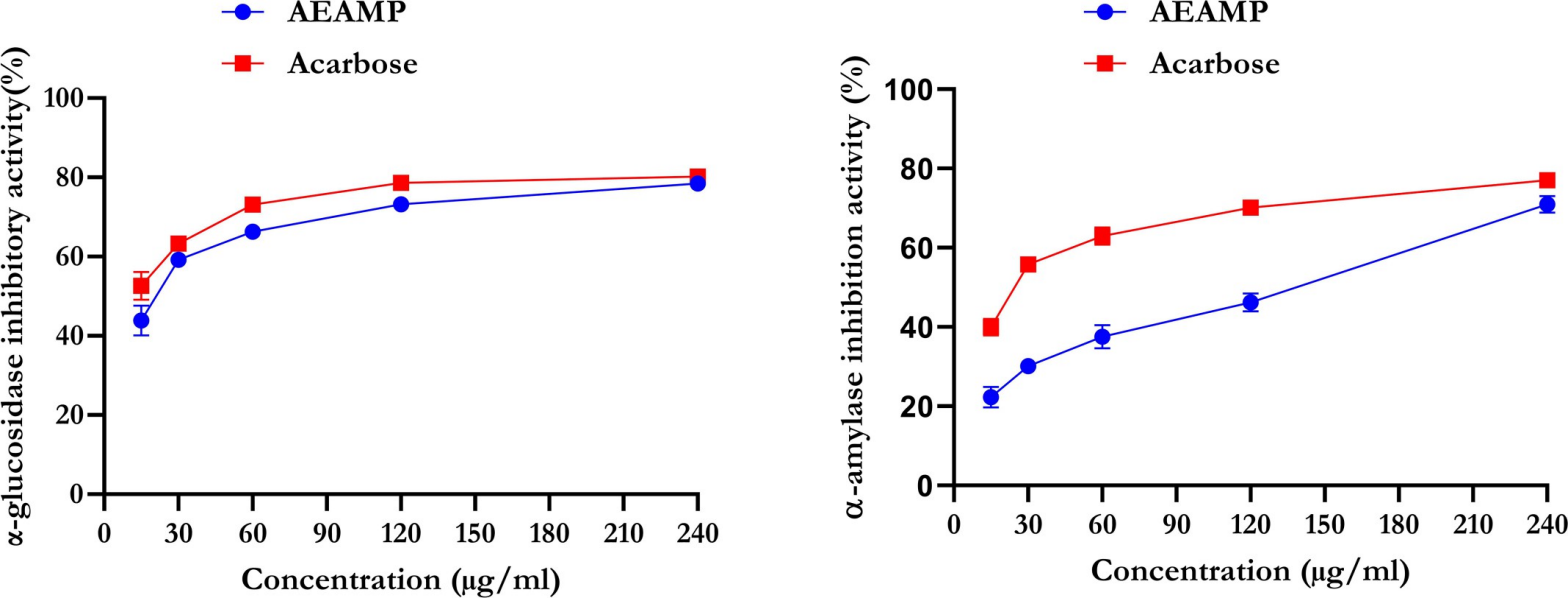
α-glucosidase and α-amylase inhibitory activities of aqueous extract of *A*. *muricata* peels. Data are expressed as mean ±SD of triplicate determinations. AEAMP: aqueous extract of *Annona muricata* peels.

Fig 2 shows that acarbose had the highest inhibitory efficacy against α-glucosidase when compared to AEAMP. AEAMP inhibited α-glucosidase at a maximum of 71.56% and acarbose at a maximum of 76.41%. Furthermore, there was a significant difference in AEAMP’s α-amylase inhibitory property (60.46%) compared to acarbose (76.41%).

### *In vivo* experimental studies

Rats administered with alloxan had an increase in FBG level. In contrast, FBG levels decreased after AEAMP administration at 6.76 mg/kg, 13.53 mg/kg, and 27.06 mg/kg b.wt. The diabetic groups treated with 27.06 mg/kg body weight had FBG levels that were comparable to the control group. At 27.06 mg/kg b.wt., the most pronounced effect was observed (Table 2). The induction of alloxan led to a significant decrease in the animals’ bodyweight (Table 3). The rats’ body weight increased after being treated with AEAMP at a dose of 26.06 mg/kg b. wt. The organ-to-body weight ratios revealed that the diabetic control pancreas values were higher than the other groups. The liver values revealed that the AEAMP-treated groups’ values were higher than the normal control rats and the metformin-treated group (Table 4).

**Table 2:** Fasting blood glucose (mg/dL) levels of alloxan-induced diabetic rats before and after oral administration of aqueous extract of *A*. *muricata* peels.

TREATMENT GROUPS	Initial FBG Value (mg/dL)	FBG Value after 48 h of induction (mg/dL)	FBG Value after 14 days of treatment (mg/dL)	FBG Value after 21 days of treatment (mg/dL)
CT	69.91 ± 1.73^c^	69.91 ± 1.73^a^	69.91 ± 1.73^a^	79.92 ± 1.94^a^
DC	66.16 ± 2.64^b^	365.38 ± 24.45^b^	393.66 ± 16.39^b^	436.14 ± 39.31^b^
DM	53.25 ± 0.92^a^	408.15 ± 21.61^c^	325.12 ± 31.21^c^	87.30 ± 2.93^a^
AM-1	64.31 ± 2.75^a^^,^^b^	491.60 ± 22.74^c^	399.27 ± 19.71^d^	115.95 ± 14. 55^c^
AM-2	66.15 ± 3.05^b^	492.01 ± 21.55^c^	375.63 ± 17.83^b^^,^^c^	110.15 ± 4.45^c^
AM-3	60.79 ± 3.36^a^^,^^b^	389.16 ± 22. 31^b^	182.70 ± 17.98^d^	88.02 ± 3.02^a^

Data are expressed as mean ± SD (n = 6). Down the column

^a-d^values with different letters are significantly different (P <0.05) from each other.

*AEAMP: Aqueous extract of *A*. *muricata* peels; CT: control group; DC: Diabetic control; DM: Diabetic metformin (30 mg/kg; AM-1: Diabetic + AEAMP (6.76 mg/kg); AM-2: Diabetic + AEAMP (13.53 mg/kg); AM-3: Diabetic + AEAMP (27.06 mg/kg).

**Table 3:** Body weight of alloxan-induced diabetic rats before and after oral administration of aqueous extract of *A*. *muricata* peels.

TREATMENT GROUPS	INITIAL WEIGHT (g)	FINAL WEIGHT (g)	% WEIGHT CHANGE
CT	243.56 ± 11.13^b^	255.46 ± 7.08^c^	11.90
DC	242.06 ± 14.79^b^	188.05 ± 24.28^b^	54.01
DM	245.33 ± 9.42^b^	210.27 ± 11.84^b^^,^^c^	35.06
AM-1	255.45 ± 3.73^a^	230.00 ± 11.34^a^	25.45
AM-2	241.17 ± 4.51^a^	231.73 ± 0.87^a^	9.44
AM-3	253.13 ± 3.19^a^	276.12 ± 4.73^a^^,^^b^	22.99

Data are expressed as mean ± SD (n = 6).

^a-c^Values with different letters along a column for a given parameter are significantly different (P <0.05) from each other.

**AEAMP**: Aqueous extract of *A*. *muricata* peels; * Weight loss (); *Weight gain (); CT: control group; DC: Diabetic control; DM: Diabetic metformin (30 mg/kg; AM-1: Diabetic + AEAMP (6.76 mg/kg); AM-2: Diabetic + AEAMP (13.53 mg/kg); AM-3: Diabetic + AEAMP (27.06 mg/kg).

**Table 4:** Organ weight and organ-body weight ratios of alloxan-induced male Wistar rats administered orally with aqueous extract of *A*. *muricata* peels.

GROUPS	PARAMETERS			
	WEIGHT OF LIVER (g)	WEIGHT OF PANCREAS (g)	LIVER-BODY WEIGHT (%)	PANCREAS-BODY WEIGHT (%)
CT	4.23±0.03^b^	0.37±0.06^c^	1.44 ± 0.16^a^	0.16 ± 0.05^a^
DC	5.04±0.20^b^	0.24±0.05^b^^c^	3.43 ± 0.08^b^	0.18 ± 0.03^a^
DM	6.47±0.17^b^	0.26±0.04^a^^b^	2.71 ± 0.06^a^	0.13 ± 0.02^a^
AM-1	4.98±0.01^a^	0.18±0.14^a^	5.43 ± 0.67^d^	0.19 ± 0.03^a^
AM-2	7.87±0.02^a^	0.14±0.02^a^	3.32 ± 0.28^b^	0.15 ± 0.04^a^
AM-3	5.04±0.09^b^	0.19±0.16^a^	4.31 ± 0.62^c^^,^^d^	0.12 ± 0.03^a^

Data are expressed as mean ± SD (n = 6).

^a-c^Values with different letters along a column for a given parameter are significantly different (P <0.05) from each other.

*AEAMP: Aqueous extract of *A*. *muricata* peels; CT: control group; DC: Diabetic control; DM: Diabetic metformin (30 mg/kg; AM-1: Diabetic + AEAMP (6.76 mg/kg); AM-2: Diabetic + AEAMP (13.53 mg/kg); AM-3: Diabetic + AEAMP (27.06 mg/kg).

Serum insulin and HOMA-β levels decreased significantly in diabetic untreated rats, whereas HOMA-IR levels increased (Table 5). The administration of AEAMP at 6.76 mg/kg, 13.53 mg/kg, and 27.06 mg/kg increased these parameters while decreasing HOMA-IR values. When AEAMP and metformin were administered to diabetic rats, the activities of GST, CAT, SOD, and GSH levels in the liver (Table 6) and pancreas (Table 7) were higher than in untreated rats. MDA levels were, however, depleted after administration of metformin and AEAMP, contrary to the increased levels in diabetic rats. The results were observed to be dose-dependent.

**Table 5:** Serum insulin levels, HOMA-IR and HOMA-β scores of alloxan-induced diabetic rats after oral administration of aqueous extract of *A*. *muricata* peels.

GROUPS	PARAMETERS		
	INSULIN (U/l)	HOMA-IR	HOMA-β
CT	10.92 ± 0.11^e^	2.09 ± 0.30^a^	47.14 ± 1.39^e^
DC	5.84 ± 0.18^a^	6.34 ± 0.72^c^	1.44 ± 0.35^a^
DM	8.09 ± 0.06^b^	1.74 ± 0.71^a^	30.01 ± 0.86^c^^,^^d^
AM-1	8.52 ± 0.12^b^	2.21 ± 0.28^a^	28.12 ± 4.93^c^
AM-2	9.02 ± 0.04^c^	4.68 ± 0.11^b^	11.98 ± 0.28^b^
AM-3	10.35 ± 0.37^d^	2.24 ± 0.75^a^	39.02 ± 1.56^d^^,^^e^

Data are expressed as mean ± SD (n = 6).

^a-e^Values with different letters along a column for a given parameter are significantly different (P <0.05) from each other.

*AEAMP: Aqueous extract of *A*. *muricata* peels

*HOMA-IR (Homeostatic model assessment of insulin resistance): [(Fasting serum insulin in U/l *fasting blood glucose in mmol/l)/22.5]

*HOMA-β (Homeostatic model assessment of β-cell function: [(Fasting serum insulin in U/l *20/fasting blood glucose in mmol/l-3.5)]

*Conversion factor: Insulin (1U/l = 7.174pmol/l); CT: control group; DC: Diabetic control; DM: Diabetic metformin (30 mg/kg; AM-1: Diabetic + AEAMP (6.76 mg/kg); AM-2: Diabetic + AEAMP (13.53 mg/kg); AM-3: Diabetic + AEAMP (27.06 mg/kg).

**Table 6:** Hepatic antioxidant markers of alloxan-induced diabetic rats after oral administration of aqueous extract of *A*. *muricata* peels.

GROUPS	PARAMETERS				
	MDA (nmol/mg protein)	CAT (U/mg protein)	SOD (U/mg protein)	GSH (μmol/mg tissue)	GST (U/mg protein)
CT	1.21 ± 0.01^a^	6.14±0.02^b^	10.05 ± 0.01^e^	20.27 ± 0.04^d^	11.22 ± 0.73^b^
DC	6.32 ± 2.48 ^b^	1.24±0.12^a^	3.12 ± 0.02^a^	8.18 ±0.08 ^a^	5.18 ± 0.03^a^
DM	3.26 ± 2.22^c^	3.06±0.03^c^	6.66 ± 0.01^b^	15.27 ± 0.03^b^	9.21 ± 0.12^c^
AM-1	3.03 ± 1.65^c^	3.61±0.72^c^	6.78 ± 0.02^b^	15.24 ± 0.01^b^	8.16 ± 0.05^d^
AM-2	1.26 ± 2.22^b^	5.69±0.54^b^^,^^d^	8.65 ± 0.04^c^	18.25 ± 0.01^c^	9.64 ± 0.23^e^
AM-3	1.06 ± 0.04 ^b^	5.12±0.06^b^^,^^d^	9.61 ± 0.01^d^^,^^e^	21.28 ± 0.02^e^	10.19 ± 0.12^b^^,^^f^

Data are expressed as mean ± SD (n = 6).

^a-f^Values with different letters along a column for a given parameter are significantly different (P <0.05) from each other.

*AEAMP: Aqueous extract of *A*. *muricata* peels; *MDA: Malondialdehyde, *CAT: Catalase, *SOD: Superoxide dismutase, *GPX: Glutathione peroxidase; *GSH: Reduced Glutathione. *GST: Glutathione s-transferase; CT: control group; DC: Diabetic control; DM: Diabetic metformin (30 mg/kg; AM-1: Diabetic + AEAMP (6.76 mg/kg); AM-2: Diabetic + AEAMP (13.53 mg/kg); AM-3: Diabetic + AEAMP (27.06 mg/kg).

**Table 7:** Pancreatic antioxidant markers of alloxan-induced diabetic rats after oral administration of aqueous extract of *A*. *muricata* peels.

GROUPS	PARAMETERS				
	MDA (nmol/mg protein)	CAT (U/mg protein)	SOD (U/mg protein)	GSH (μmol/mg tissue)	GST (U/mg protein)
CT	1.62 ± 0.14^c^	10.21 ± 0.07^a^	6.24 ± 0.01^a^	8.11 ± 0.21^e^	11.02 ± 0.51^e^
DC	3.46 ± 0.06^d^	1.15 ± 0.02^b^	0.14 ± 0.01^b^	3.16 ± 0.26^a^	0.12 ± 0.01^a^
DM	1.65 ± 0.58^c^	8.11 ± 0.04^c^	3.01 ± 0.42^c^	7.18 ± 0.20^d^	8.13 ± 0.02^c^
AM-1	1.05 ± 0.01^a^	8.07 ± 0.01^c^	3.06 ± 0.01^c^	5.17 ± 0.02^b^	6.31 ± 0.10^b^
AM-2	1.04 ± 0.21^a^	8.13 ± 0.02^c^	4.44 ± 0.06^d^	6.18 ± 0.20^c^	6.12 ± 0.02^b^
AM-3	1.42 ± 0.13^b^	11.31 ± 0.16^d^	5.36 ± 0.06^a^^,^^e^	8.18 ± 0.21^e^	9.24 ± 0.10^d^

Data are expressed as mean ± SD (n = 6).

^a-e^Values with different letters along a column for a given parameter are significantly different (P <0.05) from each other.

*AEAMP: Aqueous extract of *A*. *muricata* peels; *CAT: Catalase; *SOD: Superoxide dismutase; *GPX: Glutathione peroxidase; *GSH: Reduced Glutathione; *GST: Glutathione s-transferase; *MDA: Malondialdehyde; CT: control group; DC: Diabetic control; DM: Diabetic metformin (30 mg/kg; AM-1: Diabetic + AEAMP (6.76 mg/kg); AM-2: Diabetic + AEAMP (13.53 mg/kg); AM-3: Diabetic + AEAMP (27.06 mg/kg).

**Fig 3:**
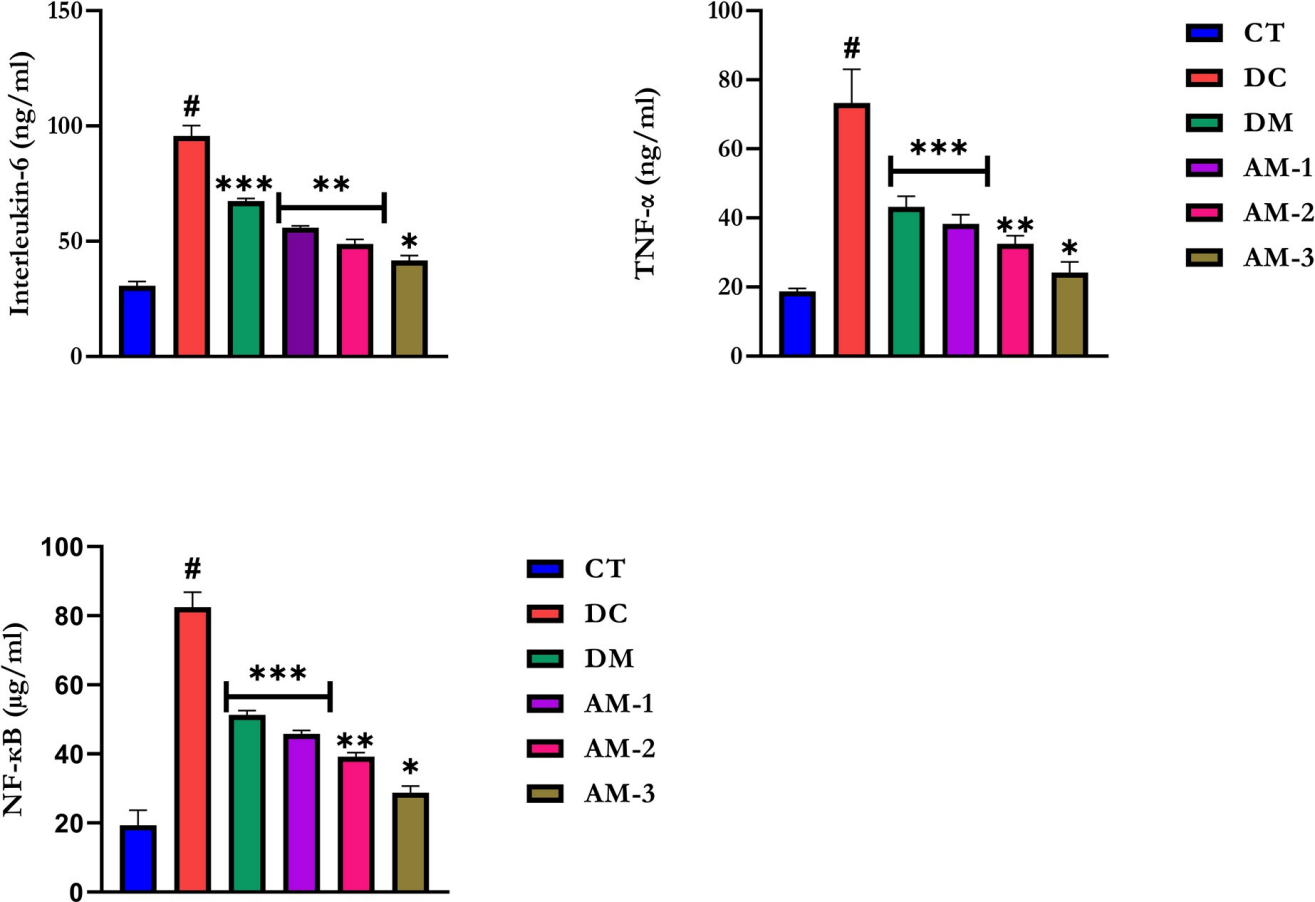
Serum interleukin-6, tumor necrosis factor-α, and nuclear factor-kappa B of alloxan-induced diabetic rats orally administered AEAMP. Data are expressed as mean ±SD (n = 6); AEAMP: aqueous extract of *Annona muricata* peels; CT: control group; DC: Diabetic control; DM: Diabetic metformin; AM-1: Diabetic + AEAMP (6.76 mg/kg); AM-2: Diabetic + AEAMP (13.53 mg/kg); AM-3: Diabetic + AEAMP (27.06 mg/kg); IL-6: Interleukin-6; TNF-α: Tumor necrosis factor-alpha; NF-κB: Nuclear factor-kappa B; **#**: significantly different from normal control (p < 0.05); * is significant at p< 0.05 and ** is significant at p< 0.01, *** is significant at p< 0.001 versus diabetic control.

Alloxan administration raised TG, CRI, AI, VLDL-c, LDL-c, and total cholesterol (TC) concentrations while decreasing HDL-c levels (Table 8). AEAMP at 6.76 mg/kg, 13.53 mg/kg, and 27.06 mg/kg decreased the levels of these parameters when compared to diabetic rats. In contrast, AEAMP dosages increased serum HDL-c levels in diabetic rats. This is similar to metformin treatment, which resulted in lowering the parameters. Alloxan induction caused a decrease in the amounts of liver glycogen and the glycolytic enzyme, hexokinase, while administration of AEAMP and metformin increased these parameters (Table 9). Furthermore, G6P and F-1,6-BP activities were increased in diabetics untreated while treatment with AEAMP and metformin resulted in a decrease in activity (Table 9). In comparison to control rats, alloxan administration increased serum concentrations of IL-6, TNF-α, and NF-κB (Fig 3). AEAMP, like metformin, reduced IL-6, TNF-α, and NF-κB levels in diabetic rats. AEAMP at 27.06 mg/kg b.wt demonstrated the most pronounced reversal of the increases observed.

**Table 8:** Lipid profile of alloxan-induced diabetic rats after oral administration of aqueous extract of *A*. *muricata* peels seeds.

GROUPS	PARAMETERS						
	TC (mmol/l)	HDL-c (mmol/l)	TG (mmol/l)	VLDL-c (mmol/l)	LDL-c (mmol/l)	AI	CRI
CT	36.21 ± 2.32^a^	29.60 ± 0.67 ^d^	30.95 ± 2.54 ^a^	6.19 ± 0.51 ^a^	0.42 ± 1.57 ^a^	35.21 ± 2.32 ^a^	1.22 ± 0.06 ^a^
DC	99.82 ± 1.16^e^	6.76 ± 0.68 ^a^	83.68 ± 0.16 ^e^	16.73 ± 0.03 ^e^	76.31 ± 1.65^f^	98.82 ± 1.16 ^e^	15.56 ± 2.05 ^b^
DM	76.05 ± 1.55^d^	21.39 ± 0.36 ^b^	63.49 ± 0.43 ^d^	12.69 ± 0.08 ^d^	41.96 ± 1.76^e^	75.05 ±1.55 ^d^	3.56 ± 0.11 ^a^
AM-1	53.29 ± 0.46^c^	23.20 ± 0.36 ^b^	47.98 ± 0.35 ^c^	9.59 ± 0.70 ^c^	20.49 ± 0.74^d^	52.29 ± 0.46 ^c^	2.30 ± 0.05 ^a^
AM-2	46.45 ± 0.92^b^	26.10 ± 0.11 ^c^	43.45 ± 0.86^b^^c^	8.69 ± 0.17^b^^c^	11.66 ± 1.89^c^	45.45 ± 0.96 ^b^	1.78 ± 0.04 ^a^
AM-3	41.70 ± 0.50^a^^b^	27.55 ± 0.45^c^^d^	38.47 ± 1.65 ^b^	7.69 ± 0.33 ^b^	6.45 ± 0.31^b^	40.70 ± 0.50^a^^b^	1.51 ± 0.27 ^a^

Data are expressed as mean ± SD (n = 6).

^a-f^Values with different letters along a column for a given parameter are significantly different (P <0.05) from each other.

*AEAMP: Aqueous extract of *A*. *muricata* peels *TC (Total cholesterol); TG(Triglyceride); *HDL-c (High density lipoprotein-cholesterol); *AI (Atherogenic index): [(TC-HDL-c)/HDL-c]; *CRI (Coronary index): [(TC(mg/dl)/HDL-c(mg/dl)]; *VLDL-c (Very low density lipoprotein-cholesterol): [TG/5]; *LDL-c (Low density lipoprotein-cholesterol): [TC-HDL-(TG/5)]; CT: control group; DC: Diabetic control; DM: Diabetic metformin (30 mg/kg; AM-1: Diabetic + AEAMP (6.76 mg/kg); AM-2: Diabetic + AEAMP (13.53 mg/kg); AM-3: Diabetic + AEAMP (27.06 mg/kg).

**Table 9:** Hepatic glycogen and carbohydrate metabolizing enzyme levels after oral administration of aqueous extract of *A*. *muricata* peels.

TREATMENT GROUPS	HEPATIC GLYCOGEN^α^	HEXOKINASE^β^	FRUCTOSE-1,6-BISPHOSPHATASE^γ^	GLUCOSE-6-PHOSPHATASE^γ^
CT	68.44 ± 3.43^c^	1.30 ± 0.04^b^^c^	0.64 ± 0.14^a^	44.34 ± 3.01^c^
DC	9.31 ± 0.47^a^	0.21 ± 0.04^a^	4.35 ± 0.61^b^	143.13 ±11.27^e^
DM	55.52 ± 3.45^b^^,^^d^	1.79 ± 0.28^c^	1.41 ± 0.13^a^	70.61 ± 0.31^d^
AM-1	46.23 ± 1.60^b^	1.01 ± 0.01^b^	1.04 ± 0.17^a^	25.90 ± 0.17^a^
AM-2	68.91 ± 3.75^c^	1.15 ± 0.02^b^	0.79 ± 0.12^a^	26.02 ± 0.23^a^
AM-3	69.08 ± 3.67^c^	1.13 ± 0.12^b^^c^	0.62 ± 0.16^a^	39.87 ± 0.24^b^^,^^c^

Data are expressed as mean ± SD (n = 6).

^a-e^Values with different letters along a column for a given parameter are significantly different (P <0.05) from each other.

*AEAMP: Aqueous extract of *A*. *muricata* peels

*^α^: Unit for glycogen (mg of glucose/g of wet tissue)

* ^β^: Unit for hexokinase (μmole glucose-6-phosphate formed/min/mg protein)

*^γ^: Unit for fructose-1,6-bisphosphatase and glucose-6-phosphatase (μmole phosphate liberated/min/mg protein); CT: control group; DC: Diabetic control; DM: Diabetic metformin (30 mg/kg; AM-1: Diabetic + AEAMP (6.76 mg/kg); AM-2: Diabetic + AEAMP (13.53 mg/kg); AM-3: Diabetic + AEAMP (27.06 mg/kg).

#### Gene expression

**Fig 4:**
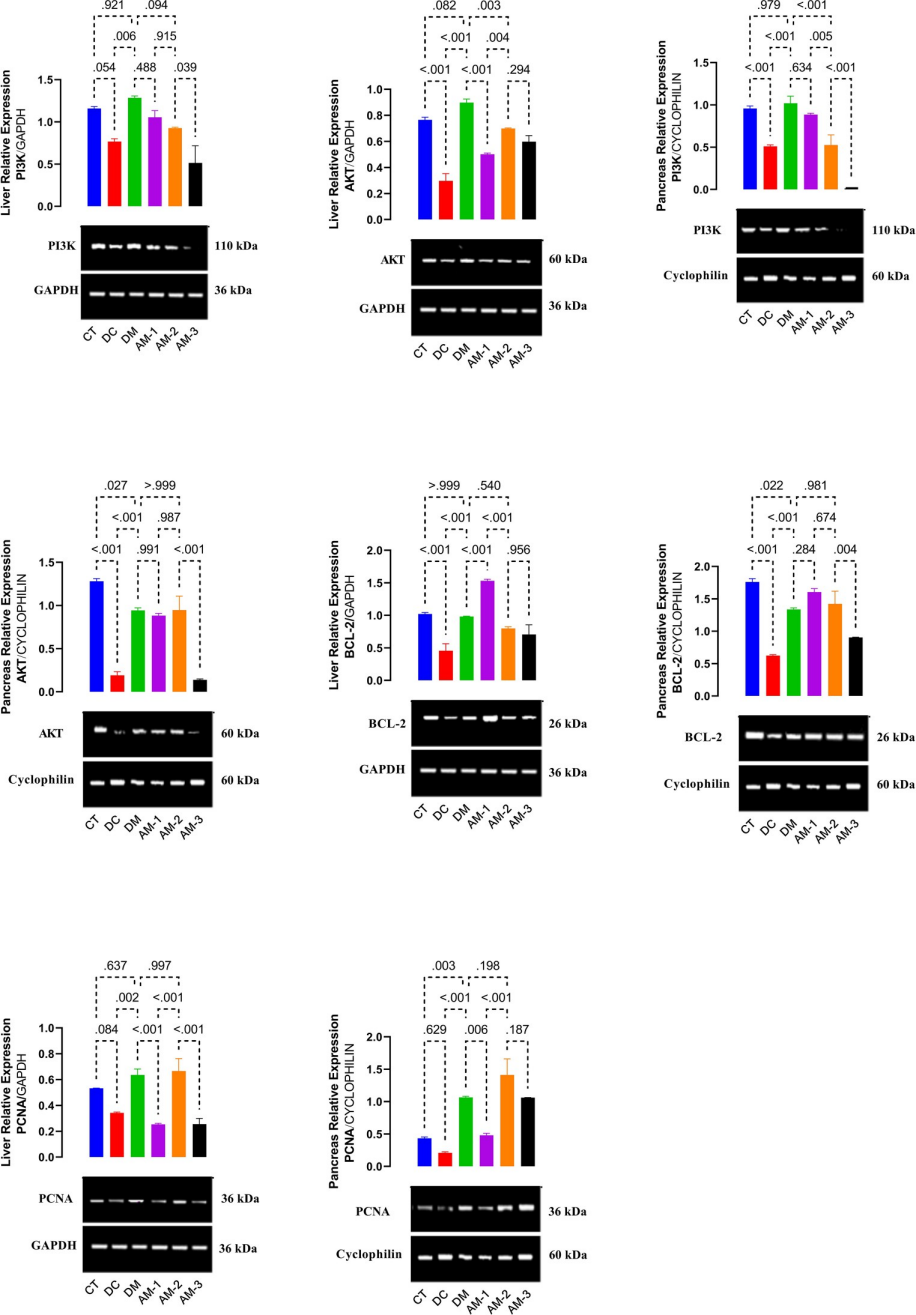
Effect of AEAMP on PI3K, AKT, Bcl2, and PCNA in liver and pancreas of diabetic rats examined by RT-PCR. GAPDH and cyclophilin were used as loading control for liver and pancreas PI3K/ATK, Bcl2, and PCNA. Data are expressed as mean ±SD (n = 6); AEAMP: aqueous extract of A. *muricata* peels; PCNA: proliferating cell nuclear antigen; CT: control group; DC: Diabetic control; DM: Diabetic metformin; AM-1: Diabetic + AEAMP (6.76 mg/kg); AM-2: Diabetic + AEAMP (13.53 mg/kg); AM-3: Diabetic + AEAMP (27.06 mg/kg).

Fig 4 depicts the PI3K, AKT, Bcl-2, and PCNA mRNA levels in the liver and pancreas. Liver PI3K, liver PCNA and pancreas PCNA were not significantly different in untreated diabetic rats when compared to normal rats suggesting alloxan induction of diabetes did not downregulate the mRNA expression of these genes. The expression of PI3K was shown to be down-regulated in the pancreas of diabetic rats. Diabetic rats, on the other hand, had reduced levels of mRNA expression for AKT, Bcl-2, and PCNA. Treatment with 6.76, 13.53, and 27.06 mg/kg of AEAMP and metformin upregulated pancreas PI3K and AKT mRNA levels, as well as Bcl-2 both in liver and pancreatic tissues (Fig 4).

#### Histological examination of the pancreas

**Fig 5:**
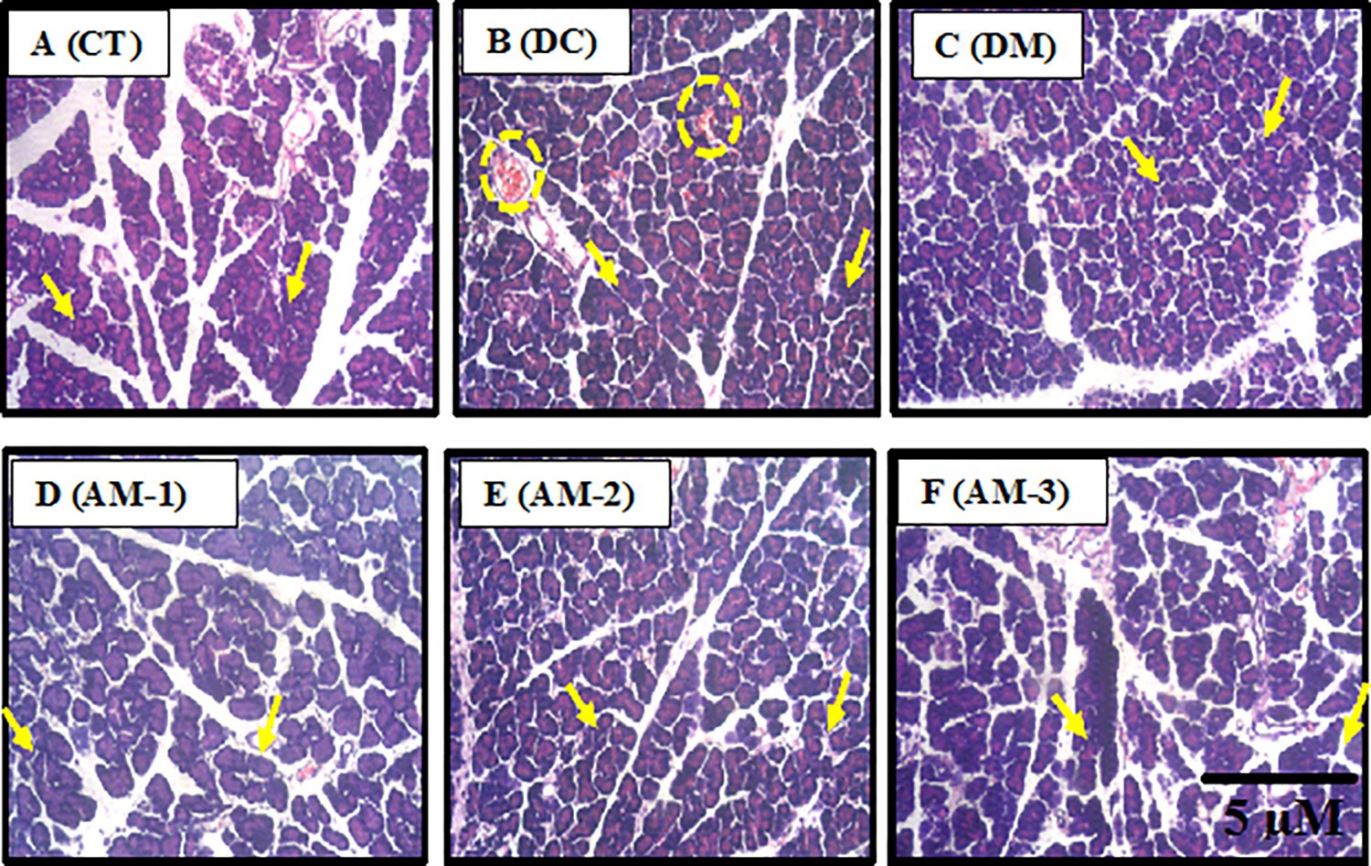
Cross section of the pancreas of rats after administration of aqueous extract of *A*. *muricata* peels. MG X100; H & E stain. (A) CT: control group showing normal histomorphological presentation of the islet cells with characteristic normal staining intensity. There are no histopathological alterations (yellow arrow); (B) DC: Diabetic control showing normal general histomorphological presentation with conspicuous metallic deposit (yellow circles) suggesting possible mild pathological alteration, mild infiltration of inflammatory cells; (C) DM: Diabetic metformin; (D) AM-1: Diabetic + AEAMP (6.76 mg/kg); (E) AM-2: Diabetic + AEAMP (13.53 mg/kg); (F) AM-3: Diabetic + AEAMP (27.06 mg/kg) shows the restoration of the islet of Langerhans (IL) (yellow arrows).

Fig 5 shows the histological evaluation of the pancreas of experimental animals after the induction of alloxan and administration of AEAMP. In contrast to the normal architecture displayed by the pancreas of normal control rats, the pancreas of alloxan-induced rats had significant necrosis, inflammatory infiltrates, and nuclear pycnosis. The administration of metformin and AEAMP decreased the occurrence of these changes in the pancreas of rats and revealed near-normal architecture comparable to the alloxan alone rats. It also improves and restores the impaired pancreatic islets cells at doses investigated.

## Discussion

The need for qualitatively extensive research for the advancement of effective antidiabetic agents is on the rise, as the increased prevalence of T1DM poses a threat to human life. Hence, plants possessing pharmacological properties are continually evaluated with the expectation of establishing plant-based antidiabetic products that are relatively safe [46, 47]. The potential of the AEAMP was revealed in this study as it was shown to elicit a decline in the heightened plasma glucose level and diabetic complications.

The use of inhibitors of the α-glucosidase and α-amylase enzymes involved in carbohydrate metabolism is a recognized technique in the management of DM for decreasing postprandial hyperglycemia [48]. Efficient control of the glycemic index in diabetes requires moderate levels of the α-amylase inhibitors and potent levels of α-glucosidase inhibitors, which aid in efficiently regulating the available dietary sugar necessary for absorption in the small intestine [49]. The inhibitory effect of AEAMP on the activity of α-glucosidase and α-amylase was demonstrated to be concentration-dependent, which could be an indication of the extract’s antidiabetic action. Similarly, some fruits, vegetables, and mushrooms, as well as phenolics, flavonoids, and their glycosides, all from plant origin, have been reported to inhibit both α-glucosidase and α-amylase [50–53].

Alloxan, a diabetogenic agent, has been shown to cause extensive selective damage to pancreatic β-cells via reactive oxygen species generation, resulting in a partial or complete defect in insulin action and hyperglycemia. In the long term, metformin has demonstrated its capability for blood glucose level reduction, mediated majorly via suppressed glucose production in the liver, with improved insulin action in peripheral tissues [54]. Fasting blood glucose is a key clinical indicator of DM. An elevation in the blood glucose levels of the diabetic rats was confirmed, validating the successful induction of DM. The reduction in fasting blood glucose levels caused by AEAMP at various dosages could be due to pancreatic β-cell restoration, and the findings are consistent with previous research indicating the extract’s potential for hypoglycemic activity [55, 56].

A decline in the body weight of diabetic rats could be associated with structural protein degradation resulting from the occurrence of diabetes as proteins play a contributory role in body weight. The reduction in body weight observed in diabetic rats could be due to a reduction in carbohydrate metabolism, increased lipid metabolism, and degradation of proteins [57]. The increase in body weight of diabetic rats given the highest dose of AEAMP could be attributed to better glycemic control via enhanced insulin secretion. The effect of AEAMP on bodyweight suggests that it has the potential to improve the weight defect, which is a feature of DM due to the decreased hyperglycemia [58]. This is in accordance with the effects of known medicinal plants, such as *Carica papaya*, *Garcinia kola*, *Muntingia calabura*, *Artemisia herba alba*, and *Dryopteris dilatata*, used in the management of diabetes [59–62].

A defect in the action of the insulin hormone is one of the outcomes of DM, usually manifested in the form of inadequate or no insulin secretion [17]. A decline in the levels of serum insulin and deterioration in the function of the β-cells of the diabetic control group were evident in this study. In the AEAMP and metformin-treated groups, a notable improvement was observed as the β-cells were restored to levels close to the normal range. Serum insulin levels may have increased in the treatment groups due to the possibility of β-cell regeneration, as indicated by the higher HOMA-β score values in the treatment group. A decrease in the HOMA-IR scores of the treatment groups in contrast to the diabetic control group might be indicative of improved insulin sensitivity aside from stimulation of glucose absorption [17, 24]. Our results are in consistent with the findings of Ojo et al. [24] who reported an increase in insulin level and decrease in HOMA-IR scores the effect of in alloxan-induced diabetic rats treated with *Persea americana* seeds.

Non-enzymatic antioxidants (MDA) and enzymatic antioxidants (CAT, SOD, GPx, GSH, and GST) play critical roles in preserving the physiological levels of H_2_O_2_ and O_2_ by enhancing the dismutation and cleaning of O_2_ radicals and peroxides created by alloxan induction [63, 64]. This investigation found that alloxan-induced stress interfered with the function of hepatic and pancreatic enzymes [65]. The observed decrease in CAT, SOD, and GST activities, as well as GSH levels in diabetic rats’ liver and pancreas, could be attributed to the reduction of alloxan to dialuric acid, resulting in the production of free radicals [66]. AEAMP increased the activities of these enzymes, which reduced the disparity between ROS generation and the activities of CAT, SOD, and GST in diabetic rats. This could be because AEAMP contains phenolics and flavonoids, both of which have been shown to have antioxidant properties. Our results correlate with the reports documented by Okoduwa et al. [46] who reported that *Ocimum gratissimum* leaf fractions improved the antioxidant activities in type-2 diabetes rat model.

The phenomenon of dyslipidemia occurring in DM has been described by alterations in plasma lipoprotein regulated by the inadequacy of the pancreatic β-cells and hyperglycemia. Insulin modulates the activity of lipoprotein at specific levels, such as gene expression and protein synthesis. Hence, a decline in lipoprotein is observed in the presence of insulin resistance occurring in a diabetic state, resulting in increased triglyceride (TG), VLDL-c, and decreased HDL-c [67]. The observed levels of VLDL-c, triglycerides (TG), LDL-c, and total cholesterol (TC) observed in the diabetic control group corroborate prior reports [62, 67]. The elevated atherogenic index and LDL-c are suggestive of susceptibility to cardiovascular diseases [17, 24]. However, administration of AEAMP and metformin led to a significant decline in the VLDL-c, TG, LDL-c, and TC levels, with a resultant increase in the HDL-c levels. This is indicative of the potential of the extract to improve dyslipidemia due to the lipase activation required for lipid hydrolysis [68]. Our results agree with those of Ojo et al. [47] who documented that the extract of *Blighia sapida* bark improved the lipid parameters in alloxan-induced diabetic rats.

Glycogen, synthesized by glycogen synthase, is a storage disposition of glucose intracellularly within the liver, and its quantification is regarded as a crucial indication of DM [69]. Physiologically, mammals respond to elevations in blood glucose levels occurring after a meal by depositing glycogen in the liver and other adjoining tissues [70]. The amount of glycogen in the liver was shown to decrease in diabetic rats, which may be because of the absence of insulin, as DM has been reported to cause impairment of the liver’s capacity to appropriately synthesize glycogen. Oral administration of AEAMP and metformin was shown to elevate the glycogen levels significantly, and this could be owing to an improvement in the activity of insulin [69, 70]. Our findings correlate with some researchers who reported that *Cenchrus purpureus* improved glycogen levels in alloxan-induced diabetic rats [44].

Hence, the liver is the location for glucose production. The two major complementary metabolic pathways responsible for creating a balance for the glucose deposit in the body, which is usually identified by a limited or complete defect in insulin activity, are gluconeogenesis and glycolysis. These pathways play crucial roles during disorders in the metabolism of glucose, resulting in elevated glucose levels. Insulin aids in preventing hyperglycemia partly by subduing glycogenolysis and gluconeogenesis while promoting glycogenesis [70]. Hexokinase, a key enzyme in glucose catabolism, is responsible for mediating phosphorylation of glucose to glucose-6-phosphate. Impairment of glucose oxidation through glycolysis could occur as a result of decreased hexokinase activity, leading to a decline in the production of ATP and hyperglycemia [69]. Hexokinase activity was detected to decline in the diabetic control group, with a notable increase after the administration of AEAMP and metformin. Suppression of blood glucose levels is suggested to arise owing to the acceleration of the glycolytic pathway [70]. The enhanced activity by AEAMP can expedite glucose use for energy production [24, 44, 62].

Glucose-6-phosphatase (G-6-Pase) is an enzyme that is essential in the maintenance of blood glucose homeostasis and delivers glucose during hyperglycemia. A decrease in G-6-Pase activity causes metabolic alterations characterized by low blood glucose triggered by cAMP. In diabetes, insulin insufficiency raises G-6-Pase activity, resulting in a higher blood glucose level. In this study, AEAMP and metformin were found to reduce G-6-Pase activity in diabetic rats. G-6-Pase activity decreases glucose synthesis [24, 71].

In a diabetic state, fructose-1,6-bisphosphatase (F-1,6-BPase) is essential for glucose release into circulation. Its activity was found to increase in diabetic rats, most likely due to a lack of insulin. Treatment with AEAMP and metformin aided in the lowering of excessive F-1,6-BPase levels in diabetic rats. This enzyme’s activity may be altered by AEAMP and metformin due to their reduction of glycolytic and glucogenic pathways [44, 70].

The flavonoids (luteolin, resorcinol, cyanidin, vanillin, and quercetin) and benzoic acid were compounds identified to be present in AEAMP. These compounds occur abundantly in plants and have been established to exhibit numerous therapeutic activities, namely antioxidant, antiviral, antifungal, anticancer, anti-inflammatory, antimicrobial, anti-allergic, analgesic, neuroprotective, antidiabetic, antihypertensive, cardioprotective, anti-obesity, antitumor, immunomodulatory, chemopreventive, as well as regulation of the PI3K/AKT signaling pathway, amongst others [72–76]. Therefore, the therapeutic activities exhibited by AEAMP, as evidenced by the results, could be because of these compounds.

**Fig 6:**
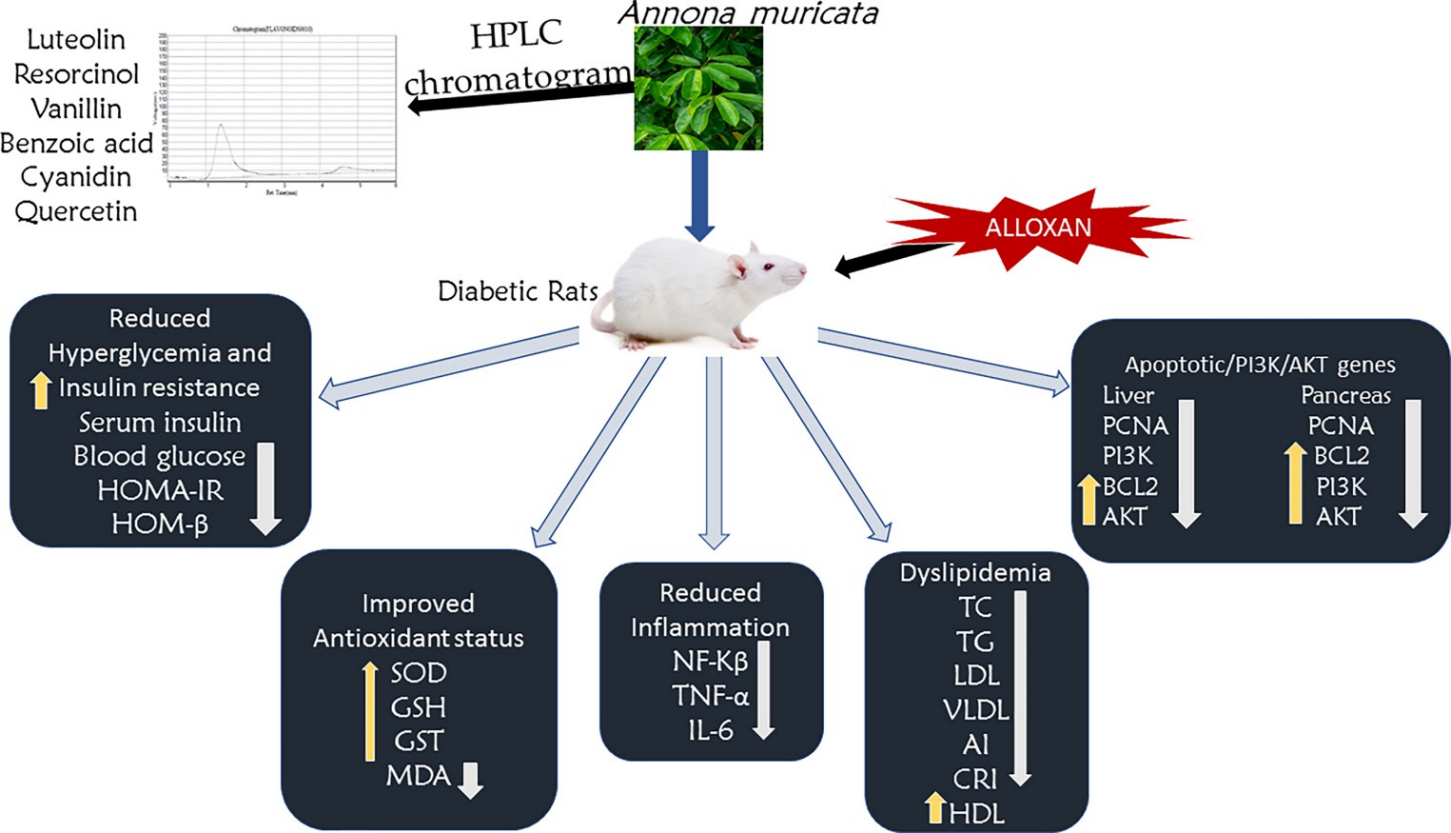
Schematic illustration of key findings of the present study. The proposed mechanism of action of *A*. *muricata* peels in diabetic rats on improving insulin binding and increase glucose metabolism. *A*. *muricata*, upregulates mRNA expression of the pancreatic PI3K, Akt, and Bcl2 genes and liver Akt, and Bcl2 genes while liver PI3K, PCNA, and pancreas PCNA were not downregulated by alloxan. This leads to increase in the insulin sensitivity and reduce the blood glucose.

The PI3K/Akt pathway appears to be a key player in mediating cell survival in a variety of situations. A number of signaling cascades, such as the phosphatidylinositol-3-OH kinase (PI3K)/Akt ([also known as protein kinase B]), the Ras-mitogen-activated protein kinase (MAPK), and the cAMP/protein kinase A (PKA) pathways, are activated by trophic factors such as NGF, insulin-like growth factor I, or BDNF. These aforementioned pathways help cells survive in specific circumstances [77]. According to Ojo *et al*. [24], the PI3K/AKT pathway is important in carbohydrate metabolism, which is required for glucose uptake enhanced by insulin in the liver [24, 78–80]. By phosphorylation, AKT activation enhances cell survival [81]. Insulin action is facilitated via the stimulation of PI3K and its effectors. AEAMP could halt oxidative injury caused by alloxan via an anti-apoptotic mechanism and boost the expression of phosphorylation of AKT [82]. AEAMP restored the decreased levels of PI3K and AKT in diabetic rats (Fig 6). This is in agreement with the study reported on the antidiabetic activity of avocado seeds in diabetic rats via upregulation of PI3K/AKT [24].

BCL-2, an anti-apoptotic molecule, is a key player involved in cell death signaling. Alloxan induction caused a lower expression of BCL-2 in hepatic and pancreatic tissues, whereas AEAMP significantly changed the equilibrium of anti-apoptotic molecules and averted apoptosis in these tissues at 6.76 and 13.53 mg/kg, respectively, as did metformin. This is consistent with the studies reported on the effect of *Cenchrus purpureus* in diabetic rats and the comparison of streptozotocin-induced diabetic and insulin resistant effects on spermatogenesis with proliferating cell nuclear antigen (PCNA) immunostaining of adult rat testis [44, 83].

At the start of cell propagation, proliferating cell nuclear antigen (PCNA), a vital proliferation marker, facilitates DNA polymerase. In addition to antibodies against foreign substances, PCNA plays an important role in the eukaryotic cell cycle [24, 44, 82, 84]. PCNA mRNA expression was found to be upregulated in normal rat hepatocytes and pancreatic tissues but downregulated in diabetic rats. AEAMP increased PCNA expression in diabetic rats’ liver and pancreatic tissues. This is consistent with earlier studies reported on the effect of *Persea americana* seeds and *Cenchrus purpureus* on PCNA expression in diabetic rats [24, 44].

## Conclusions

Taken together, AEAMP reduces insulin resistance in diabetic rats. In diabetic rats, the probable molecular processes include an increase in glucose absorption, improved hexokinase activity, and a reduction in pancreatic β-cell apoptosis via the PI3K/Akt gene upregulation (Fig 6). Because it reveals a regulated high blood glucose level and other related biochemical parameters, AEAMP could be a valuable source of antidiabetic drugs. Liver PI3K, liver PCNA and pancreas PCNA were not significantly different in untreated diabetic rats when compared to normal rats suggesting alloxan induction of diabetes did not downregulate the mRNA expression of these genes. AEAMP significantly up-regulated expression of AKT and Bcl2 in the liver and pancreatic tissue.

## Supporting information

S1 File (PDF)

## References

[1] Khan MAB, Hashim MJ, King JK, Govender RD, Mustafa H, Al Kaabi J. Epidemiology of Type 2 Diabetes—Global Burden of Disease and Forecasted. Trends J Epidemiol Glob Health. 2020; 10: 107–111. doi: 10.2991/jegh.k.191028.001 32175717 PMC7310804

[2] International Diabetes Federation. *IDF diabetes atlas*. IDF http://www.diabetesatlas.org/component/attachments/?task=download&id=116. Accessed on 2015.

[3] Melmed S, Koenig R, Rosen C, Auchus R, Goldfine A. Williams textbook of endocrinology E-Book. 14^th^ ed. Elsevier Health Sciences, 2019.

[4] Mobasseri M, Shirmohammadi M, Amiri T, Vahed N, Fard HH, Ghojazadeh M. Prevalence and incidence of type 1 diabetes in the world: a systematic review and meta-analysis. Health promotion perspectives, 2020; 10: 98. doi: 10.34172/hpp.2020.18 32296622 PMC7146037

[5] Katsarou A, Gudbjörnsdottir S, Rawshani A, Dabelea D, Bonifacio E, Anderson BJ, et al. Type 1 diabetes mellitus. Nature reviews Disease primers, 2017; 3: 1–7. doi: 10.1038/nrdp.2017.16 28358037

[6] Basina M, Maahs DM. Age at type 1 diabetes onset: a new risk factor and call for focused treatment. Lancet. 2018; 392: 453–4. doi: 10.1016/S0140-6736(18)31811-7 30129445

[7] International Diabetes Federation (IDF), *IDF Diabetes Atlas*, 9th edn. 2019: Brussels, Belgium: International Diabetes Federation, 2019.

[8] Rashid T, et al. Origin and history of immunogenetics. InA Molecular Approach to Immunogenetics, 2022; 1–19. Academic Press.

[9] Osadebe PO, Odoh EU, Uzor PF. The search for new hypoglycemic agents from plant. Afr J Pharma Pharmacol. 2014; 8: 292–303.

[10] Weinstock RS. Patient education: Self-monitoring of blood sugar in diabetes (Beyond the Basics). Portal Uptpdate [08/05/2019]. *Disponível em*:*< http://twixar*. *me/KGP1>*. *Acesso em* 10, no. 10 (2019).

[11] Brunerova L, Brož J. Oral antidiabetic drugs in treatment of type 1 diabetes mellitus. Vnitr̆ní Lékar̆ství. 2016; 62: 998–1003. 28139129

[12] Gitelman SE, Bundy BN, Ferrannini E, Lim N, Blanchfield JL, DiMeglio LA, et al. Imatinib therapy for patients with recent-onset type 1 diabetes: a multicentre, randomised, double-blind, placebo-controlled, phase 2 trial. The Lancet Diabetes & Endocrinol. 2021; 9: 502–14. doi: 10.1016/S2213-8587(21)00139-X 34214479 PMC8494464

[13] Arumugam G, Manjula P, Paari N. A review: Anti diabetic medicinal plants used for diabetes mellitus. J Acute Dis. 2013; 2: 196–200.

[14] Mohammed A, Tajuddeen N. Antidiabetic compounds from medicinal plants traditionally used for the treatment of diabetes in Africa: A review update (2015–2020). South Afr J Bot. 2022; 146: 585–602.

[15] Kazi S. Use of traditional plants in diabetes mellitus. Int J Pharma. 2014; 4: 283–289.

[16] Hashmat I, Azad H, Ahmed A. Neem (Azadirachta indica A. Juss)-A nature’s drugstore: an overview. Int Res J Biol Sci. 2012; 1: 76–79.

[17] Ojo OA, Ojo AB, Ajiboye BO, Oyinloye BE, Imiere O, Adeyonu, O. Ameliorative potentials of Blighia sapida K.D. Koenig bark against pancreatic-cell dysfunction in alloxan-induced diabetic rats. J. Complement. Integr. Med. 2017; 14(3) article number 20160145:/j/jcim.2017.14.issue-3/jcim-2016-0145/jcim-2016-0145.xml. 10.1515/jcim-2016-0145 28306534

[18] Nazeerullah K, Sunil K, Pal SR, Dhankhar N, Modi K, Nagar M. A pharmacognostic and pharmacological overview on *Caesalpinia bonducella*. Res J Pharma Biol Chem Sci. 2012; 3: 440–496.

[19] Moghadamtousi SZ, Fadaeinasab M, Nikzad S, Mohan G, Ali HM, Kadir HA. *Annona muricata* (Annonaceae): a review of its traditional uses, isolated acetogenins and biological activities. Int J Mole Sci. 2015; 16: 15625–58. 10.3390/ijms160715625 PMC4519917 26184167

[20] Coria-Téllez AV, Montalvo-Gónzalez E, Yahia EM, Obledo-Vázquez EN. *Annona muricata*: A comprehensive review on its traditional medicinal uses, phytochemicals, pharmacological activities, mechanisms of action and toxicity. Arabian J Chem. 2018; 11: 662–91.

[21] Justino AB, Miranda NC, Franco RR, Martins MM, Maria da Silva N, Espindola FS. *Annona muricata Linn*. leaf as a source of antioxidant compounds with in vitro antidiabetic and inhibitory potential against α-amylase, α-glucosidase, lipase, non-enzymatic glycation and lipid peroxidation. Biomed Pharmacother. 2018; 100: 83–92. 29425747 10.1016/j.biopha.2018.01.172

[22] Okolie NP, Agu KC, Eze GI. Protective effect of ethanolic leaf extract of *Annona muricata* Linn on some early events in Cycas-induced colorectal carcinogenesis in rats. J Pharma Sci Innov. 2013; 2:14–21.

[23] Agu KC, Okolie NP, Eze I, Anionye JC1, Falodun A. Phytochemical analysis, toxicity profile and hemo-modulatory properties of *Annona muricata* (soursop). Egyptian J Haematol. 2017; 42: 36–44.

[24] Ojo OA, Amanze J, Oni AP, Grant S, Iyobhebhe M, Elebiyo TC, et al, Antidiabetic activity of avocado seeds *(Persea americana Mill*.*)* in diabetic rats via activation of PI3K/AKT signaling pathway. Scientific Rep. 2022a; 12: 2919 doi: 10.1038/s41598-022-07015-8 35190649 PMC8861005

[25] Shai L.J., Masoko P. and Mokgotho M.P., Yeast alpha glucosidase inhibitory and antioxidant activities of six medicinal plants collected in Phalaborwa, South Africa. South African Journal of Botany, 2010; 76: 65–470.

[26] Nguelefack TB, Fofie CK, Nguelefack-Mbuyo EP, Wuyt AK. Multimodal alpha-Glucosidase and alpha-Amylase Inhibition and Antioxidant Effect of the Aqueous and Methanol Extracts from the Trunk Bark of *Ceiba pentandra*. Biomed. Res Int. 2020; 3:1–13. doi: 10.1155/2020/3063674 32382543 PMC7191384

[27] Nair AB, Jacob S. A simple practice guide for dose conversion between animals and human. J Basic Clin Pharm. 2016; 7: 27–31. doi: 10.4103/0976-0105.177703 27057123 PMC4804402

[28] Ibrahim MA, Islam MS. Butanol fraction of Khaya senegalensis root modulates β cell function and ameliorates diabetes-related biochemical parameters in a Type 2 diabetes rat model. Journal of Ethnopharmacology, 2014; 154: 832–838. doi: 10.1016/j.jep.2014.05.011 24846204

[29] Murat JC, Serfaty A. Simple enzymatic determination of polysaccharide (glycogen) content of animal tissues. Clinical Chemistry, 1974; 20: 1576–1577. 4473281

[30] Fredrickson DS, Levy RI, Lees RS. Fat transport in lipoproteins: an integrated approach to mechanisms and disorders. New England Journal of Medicine, 1967; 276: 34–281. doi: 10.1056/NEJM196701052760107 5333081

[31] Tietz NW. *Clinical guide to Laboratory Tests*. 3^rd^ edition, W.B.: Saunders Company, Philadelphia, 1994.

[32] Jacobs RF, Sowell MK, Moss MM, Fiser DH. Septic shock in children: bacterial etiologies and temporal relationships. Pediatr Infect Dis J. 1990; 9: 196–200. doi: 10.1097/00006454-199003000-00010 2336300

[33] Friedewald WT, Levy RI, Fredrickson DS. Estimation of the concentration of low density lipoprotein cholesterol in plasma, without use of the preparative ultracentrifuge. Clin Chem. 1972;18: 499–502. 4337382

[34] Liu CS, Lin CC, Li TC. The relation of white blood cell count and atherogenic index ratio of LDL-cholesterol to HDL-cholesterol in Taiwan school children. Acta Paediatrica Taiwanica. 1999; 40: 319–324. 10910541

[35] Boers M, Nurmohamed MT, Doelman CJ, Lard LR, Verhoeven AC, Voskuyl AE, et al. Influence of glucocorticoids and disease activity on total and high density lipoprotein cholesterol in patients with rheumatoid arthritis. Annals of the Rheumatic Dis. 2003; 62: 842–845. doi: 10.1136/ard.62.9.842 12922956 PMC1754645

[36] Wilson RD, Islam MS. Fructose-fed streptozotocin-injected rat: an alternative model for Type 2 diabetes. Pharmacol Rep. 2012; 64: 129–139. doi: 10.1016/s1734-1140(12)70739-9 22580529

[37] Livingstone C, Davis J. Review: Targeting therapeutics against glutathione depletion In diabetes and its complications. Br J Diabetes Vascular Dis. 2007; 7:258–265.

[38] Winterbourn CC. Superoxide as an intracellular radical sink. Free Rad Biol Med. 1993; 14: 85–90. doi: 10.1016/0891-5849(93)90512-s 8384151

[39] Zelko IN, Manriani YJ, Folz RJ. Superoxide dismutase multigene family: a comparison of the CuZn-SOD (SOD1), Mn-SOD (SOD2), and EC-SOD (SOD3) gene structures, evolution, and expression. Free Rad Biol Med. 2002; 33:337–349. doi: 10.1016/s0891-5849(02)00905-x 12126755

[40] Varshney R, Kale RK. Effects of Calmodulin antagonists on radiation-induced lipid peroxidation in microsomes. Int J Rad Biol. 1990;58: 733–743. 1977818 10.1080/09553009014552121

[41] Zhang Z, Jiang J, Yu P, Zeng X, Larrick J, Wang Y. Hypoglycemic and beta cell protective effects of andrographolide analogue for diabetes treatment. J Translat Med. 2009.7: p. 62–75. doi: 10.1186/1479-5876-7-62 19607676 PMC3224956

[42] Erukainure OL, Mopuri R, Oyebode OA, Koorbanally NA, Islam MS. Dacryodes edulis enhances antioxidant activities, suppresses DNA fragmentation in oxidative pancreatic and hepatic injuries; and inhibits carbohydrate digestive enzymes linked to type 2 diabetes. Biomed Pharmacother. 2017; 96: 37–47. doi: 10.1016/j.biopha.2017.09.106 28963949

[43] Gancedo JM, Gancedo C. Fructose 1, 6-bisphophatase, phosphofructokinase and glucose 6-phosphate dehydrogenase from fermenting yeast. Arc Microbiol. 1971; 76: 132–138. 10.1007/BF00411787 4324161

[44] Ojo OA, Oni AP, Grant S, Amanze J, Ojo AB, Taiwo OA, et al. Antidiabetic activity of Elephant grass (*Cenchrus purpureus* (Schumach.) Morrone) via activation of PI3K/AkT signaling pathway, oxidative stress inhibition, and apoptosis in Wistar rats. Frontiers in pharmacol. 2022b; 13: 1–15 doi: 10.3389/fphar.2022.845196 35308202 PMC8924541

[45] Drury RA, Wallington EA, Cancerson R. Carlton’s Histopathological Techniques (4th ed.). Oxford, UK: Oxford University Press. 1976. https://www.scirp.org/(S(lz5mqp453edsnp55rrgjct55))/reference/ReferencesPapers.aspx?ReferenceID=1436264.

[46] Okoduwa SI, Umar IA, James DB, Inuwa HM. Anti-diabetic potential of Ocimum gratissimum leaf fractions in fortified diet-fed streptozotocin treated rat model of type-2 diabetes. Medicines, 2017; 4: 73–94. doi: 10.3390/medicines4040073 29019956 PMC5750597

[47] Ojo OA, Ojo AB, Ajiboye BO, Imiere O, Oyinloye BE. Antihyperlipidemic activities and hematological potentials of ethanol extract of Blighia sapida Koenig bark in alloxan-induced diabetic rats. Serbian J Exp Clin Res. 2020a; 21: 11–17 doi: 10.2478/SJECR-2018-0042

[48] Adefegha SA, Oboh G. Inhibition of key enzymes linked to type 2 diabetes and sodium nitroprusside-induced lipid peroxidation in rat pancreas by water extractable phytochemicals from some tropical spices. Pharma Biol. 2012; 50: 857–865. doi: 10.3109/13880209.2011.641022 22480175

[49] Ojo OA, Afon AA, Ojo AB, Ajiboye BO, Oyinloye BE, Kappo AP. Inhibitory Effects of Solvent-Partitioned Fractions of Two Nigerian Herbs (Spondias mombin Linn. and Mangifera indica L.) on α-Amylase and α-Glucosidase. Antioxidants, 2018; 7: 73 doi: 10.3390/antiox7060073 29861455 PMC6025479

[50] Apostolidis E, Lee CM. In vitro potential of Ascophyllum nodosum phenolic antioxidant‐mediated α‐glucosidase and α‐amylase inhibition. J Food Sci. 2010; 75: 97–102. 10.1111/j.1750-3841.2010.01544.x 20492300

[51] Papoutsis K, Zhang J, Bowyer MC, Brunton N, Gibney ER, Lyng J. Fruit, vegetables, and mushrooms for the preparation of extracts with α-amylase and α-glucosidase inhibition properties: A review. Food Chemistry, 2021; 338: 28119. 10.1016/j.foodchem.2020.128119 33091976

[52] Sun H, Wang D, Song X, Zhang Y, Ding W, Peng X, et al. Natural prenylchalconaringenins and prenylnaringenins as antidiabetic agents: α-glucosidase and α-amylase inhibition and in vivo antihyperglycemic and antihyperlipidemic effects. J Agric Food Chem. 2017; 65: 574–1581. doi: 10.1021/acs.jafc.6b05445 28132506

[53] Cardullo N, Muccilli V, Pulvirenti L, Cornu A, Pouységu L, Deffieux D, et al. C-glucosidic ellagitannins and galloylated glucoses as potential functional food ingredients with anti-diabetic properties: A study of α-glucosidase and α-amylase inhibition. Food chem. 2020; 313: 126099. doi: 10.1016/j.foodchem.2019.126099 31927321

[54] Horakova O, Kroupova P, Bardova K, Buresova J, Janovska P, Kopecky J, et al. Metformin acutely lowers blood glucose levels by inhibition of intestinal glucose transport. Sci Rep. 2019; 9: 1–11 30992489 10.1038/s41598-019-42531-0 PMC6468119

[55] Alhassan AJ, Sule MS, Atiku MK, Wudil AM, Mohammed AS. Effects of aqueous avocado pear (Persea americana) seed extract on alloxan induced diabetes rats. Greener J Med Sci. 2012; 2: 005–011.

[56] Ejiofor CC, Ezeagu IE, Ayoola M. Hypoglycaemic and Biochemical effects of the aqueous and methanolic extract of Persea americana seeds on alloxan-induced albino rats. European J Med Plants, 2018; 26: 1–12.

[57] Yakubu MT, Uwazie NJ, Igunnu A. Anti-diabetic Activity of Aqueous Extract of Senna alata (Fabacea) Flower in Alloxan-induced Diabetic Male Rats. Cameroon J Biol Biochem Sci. 2016; 24: 7–17.

[58] Yakubu MT, Ogunro OB. Effects of aqueous extract of Fadogia agrestis stem in alloxan-induced diabetic rats. Bangladesh J Pharmacol. 2014; 9: 356–363.

[59] Ewenighi C. Estimation of glucose level and body weight in alloxan induced diabetic rat treated with aqueous extract of Garcinia kola seed. The Ulutas Medical J. 2015; 1: 26–30.

[60] Solikhah TI, Solikhah GP. Effect of Muntingia calabura L. leaf extract on blood glucose levels and body weight of alloxan-induced diabetic mice. Pharmacog J. 2021;13: 1450–1455.

[61] Sekiou O, Boumendjel M, Taibi F, Tichati L, Boumendjel A, Messarah M. Nephroprotective effect of Artemisia herba alba aqueous extract in alloxan-induced diabetic rats. J Trad Compl Med. 2021;11: 53–61. doi: 10.1016/j.jtcme.2020.01.001 33511062 PMC7817709

[62] Ajirioghene AE, Ghasi SI, Ewhre LO, Adebayo OG, Asiwe JN. Anti-diabetogenic and in vivo antioxidant activity of ethanol extract of Dryopteris dilatata in alloxan-induced male Wistar rats. Biomarkers, 2021;26: 718–725. doi: 10.1080/1354750X.2021.1990408 34612093

[63] Lekshmi RK, Rajesh R, Mini S. Ethyl acetate fraction of Cissus quadrangularis stem ameliorates hyperglycaemia-mediated oxidative stress and suppresses inflammatory response in nicotinamide/streptozotocin induced type 2 diabetic rats. Phytomed, 2015; 22: 952–960, doi: 10.1016/j.phymed.2015.06.014 26321745

[64] Ajiboye BO, Oyinloye BE, Essien PE, Onikanni SA, Ojo OA, Kappo AP. Ameliorative potential of Sterculia tragacantha aqueous extract on renal gene expression and biochemical parameters in streptozotocin-induced diabetic rats. J Pharmaceutical Investigation, 2021; 51: 103–113 doi: 10.1007/s40005-020-00506-8

[65] Ojo OA, Osukoya OA, Ekakitie L, Ajiboye BO, Oyinloye BE, Agboinghale PE, et al. Gongronema latifolium leaf extract modulates hyperglycaemia, inhibits redox imbalance and inflammation in alloxan-induced diabetic nephropathy. J Diabetes & Metabolic Disorders, 2020b;19: 469–481 doi: 10.1007/s40200-020-00533-0 32550199 PMC7270381

[66] Lenzen S. The mechanisms of alloxan- and streptozotocin-induced diabetes. Diabetologia, 2008; 51: 216–226. doi: 10.1007/s00125-007-0886-7 18087688

[67] Artha IM, Bhargah A, Dharmawan NK, Pande UW, Triyana KA, Mahariski PA, et al. High level of individual lipid profile and lipid ratio as a predictive marker of poor glycemic control in type-2 diabetes mellitus. Vascular Health and Risk Management, 2019; 15: 149–157. doi: 10.2147/VHRM.S209830 31239693 PMC6560183

[68] Oyedemi SO, Yakubu MT, Afolayan AJ. Antidiabetic activities of aqueous leaves extract of Leonotis leonurus in streptozotocin induced diabetic rats. J Med Plants Res. 2011; 5: 119–125.

[69] Ramachandran V, Saravanan R. Efficacy of asiatic acid, a pentacyclic triterpene on attenuating the key enzymes activities of carbohydrate metabolism in streptozotocin-induced diabetic rats. Phytomed. 2013; 20: 230–236. doi: 10.1016/j.phymed.2012.09.023 23102509

[70] Murali R, Srinivasan S, Ashokkumar N. Antihyperglycemic effect of fraxetin on hepatic key enzymes of carbohydrate metabolism in streptozotocin-induced diabetic rats. Biochimie, 2013; 95: 1848–1854 doi: 10.1016/j.biochi.2013.06.013 23806420

[71] Xia X, Yan J, Shen Y, Tang K, Yin J, Zhang Y, et al. Berberine improves glucose metabolism in diabetic rats by inhibition of hepatic gluconeogenesis. PLoS One, 2011; 6: e16556. doi: 10.1371/journal.pone.0016556 21304897 PMC3033390

[72] Lin S, Ramulu P, Lamoureux EL, Sabanayagam C. Addressing risk factors, screening, and preventative treatment for diabetic retinopathy in developing countries: a review. Clin Exp Ophthalmol. 2016; 44: 300–320. doi: 10.1111/ceo.12745 26991970

[73] Aziz N, Kim MY, Cho JY. Anti-inflammatory effects of luteolin: A review of in vitro, in vivo, and in silico studies. J Ethnopharmacol. 2018; 225: 342–358. doi: 10.1016/j.jep.2018.05.019 29801717

[74] Fang F, Li D, Pan H, Chen D, Qi L, Zhang R, et al., Luteolin Inhibits Apoptosis and Improves Cardiomyocyte Contractile Function through the PI3K/Akt Pathway in Simulated Ischemia/Reperfusion. Frontiers in Pharmacol. 2011; 88: 149–158. doi: 10.1159/000330068 21934351

[75] Peng J, Li Q, Li K, Zhu L, Lin X, Lin X, et al. Quercetin Improves Glucose and Lipid Metabolism of Diabetic Rats: Involvement of Akt Signaling and SIRT1. J Diabetes Res. 2017:3417306. doi: 10.1155/2017/3417306 29379801 PMC5742905

[76] Les F, Cásedas G, Gómez C, Moliner C, Valero MS, López V. The role of anthocyanins as antidiabetic agents: from molecular mechanisms to in vivo and human studies. J Physiol Biochem. 2021; 77: 109–131. doi: 10.1007/s13105-020-00739-z 32504385

[77] Rai SN, Dilnashin H, Birla H, Singh SS, Zahra W, Rathore AS, et al. The Role of PI3K/Akt and ERK in Neurodegenerative Disorders. Neurotox Res. 2019; doi: 10.1007/s12640-019-0003-y 30707354

[78] Wang N, Li T, Han P. The effect of tianmai xiaoke pian on insulin resistance through PI3-K/AKT signal pathway. J Diabetes Res. 2016:9261259. doi: 10.1155/2016/9261259 26640808 PMC4657077

[79] Nandipati KC, Subramanian S, Agrawal DK. Protein kinases: mechanisms and downstream targets in inflammation-mediated obesity and insulin resistance. Mole Cellular Biochem. 2017; 426: 27–45. doi: 10.1007/s11010-016-2878-8 27868170 PMC5291752

[80] Yu N, Fang X, Zhao D, Mu Q, Zuo J, Zhang Y, et al. Anti-diabetic effects of Jiang Tang Xiao Ke granule via PI3K/Akt signalling pathway in type 2 diabetes KKAy mice. PLoS One. 2017; 12: e0168980. doi: 10.1371/journal.pone.0168980 28045971 PMC5207690

[81] Woodgett JR. Recent advances in the protein kinase B signaling pathway. Current Opinion in Cell Biol. 2005; 17: 150–157. doi: 10.1016/j.ceb.2005.02.010 15780591

[82] Chen L, Xiang Y, Kong L, Zhang X, Sun B, Wei X, et al. Hydroxy-safflor yellow A protects against cerebral ischemia–reperfusion injury by anti-apoptotic effect through PI3K/AKT/GSK3β pathway in rat. Neurochem Res. 2013; 38: 2268–2275. doi: 10.1007/s11064-013-1135-8 23990223

[83] Zhao Y, Zhao H, Zhai X, Dai J, Jiang X, Wang G, et al., Effects of Zn deficiency, antioxidants, and low-dose radiation on diabetic oxidative damage and cell death in the testis. Toxicol Mechanisms Methods. 2013; 23: 42–47. 10.3109/15376516.2012.731437 22992206

[84] Arikawe A, Oyerinde A, Olatunji-Bello I, Obika LF, Comparison of streptozotocin-induced diabetic and insulin resistant effects on spermatogenesis with proliferating cell nuclear antigen (PCNA) immunostaining of adult rat testis. J Exp Clin Med. 2012; 29: 209–214.

