# Supplementary material for: Annona muricata L. peel extract inhibits carbohydrate metabolizing enzymes and reduces pancreatic β-cells, inflammation, and apoptosis via upregulation of PI3K/AKT genes
Source: PLoS One. 2022 Oct 27;17(10):e0276984. doi: 10.1371/journal.pone.0276984 (PMC9612462; doi:10.1371/journal.pone.0276984)
Supplement: S1 File — (PDF) [file pone.0276984.s001.pdf]

Annona muricata L. peel extract inhibits carbohydrate metabolizing enzymes and reduces pancreatic β-cells, inflammation, and apoptosis via upregulation of PI3K/AKT genes

Oluwafemi Adeleke Ojo[1*,#a], Susan Grant[1¶], Jennifer Chidubem Amanze[1¶], Abosede Itunuoluwa Oni[1¶], Adebola Busola Ojo[2], Tobiloba Christiana Elebiyo[1], Tajudeen Olabisi Obafemi[3], Damilare Iyinkristi Ayokunle[4], Akingbolabo Daniel Ogunlakin[5]

[1] Department of Biochemistry, Landmark University, Omu-Aran, Nigeria
[2] Department of Biochemistry, Ekiti State University, Ado-Ekiti, Nigeria
[3] Department of Biochemistry, Afe Babalola University, Ado-Ekiti, Nigeria
[4] Department of Pure and Applied Biology, Bowen University, Iwo, Nigeria
[5] Department of Biochemistry, Bowen University, Iwo, Nigeria
[#a] Current Address: Phytomedicine, Molecular Toxicology, and Computational Biochemistry Research Laboratory (PMTCB-RL), Department of Biochemistry, Bowen University, Iwo, Nigeria

[*]Corresponding author address: oluwafemiadeleke08@gmail.com; Tel: +2347037824647

[¶]These authors contributed equally to this work.

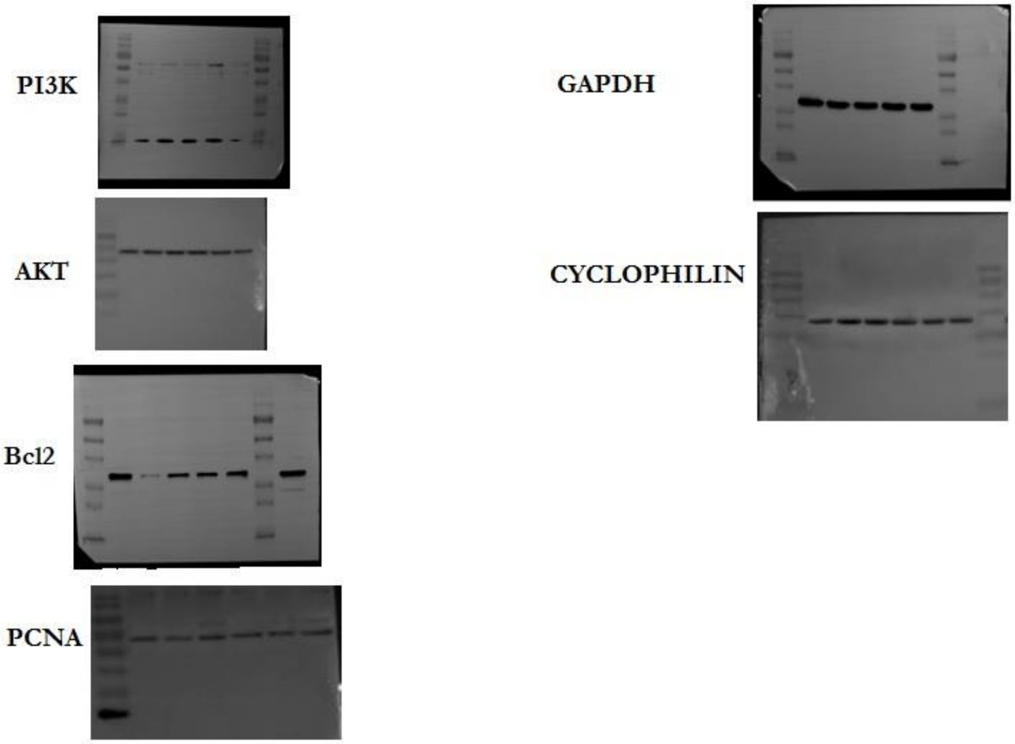

S1 Fig: Supplementary figure for the original source of blot images

PI3K mRNA Sequence (5'->3')

Forward primer GGTGCTAAGGAGGAGCACTG

Reverse primer CCATGTGGTACAGGCCAGAG

AKT mRNA Sequence (5'->3')

Forward primer AAGGACCCTACACAGAGGCT

Reverse primer AAGGTGGGCTCAGCTTCTTC

GAPDH mRNA Sequence (5'->3')

Forward primer GCATCTTCTTGTGCAGTGCC

Reverse primer GAGAAGGCAGCCCTGGTAAC

Bcl-2 mRNA Sequence (5'->3')

Forward primer GCGTCAACAGGGAGATGTCA

Reverse primer TTCCACAAAGGCATCCCAGC

PCNA mRNA Sequence (5'->3')

Forward primer AGCAACTTGGAATCCCAGAACA

Reverse primer CACAGGAGATCACCACAGCA

Cyclophilin A mRNA Sequence (5'->3')

Forward primer TGGAGAGCACCAAGACAGACA

Reverse primer TGCCGGAGTCGACAATGAT
